# Integrated health checks as a person-centred approach to systematic screening of household tuberculosis contacts: A realist-informed mixed-methods study

**DOI:** 10.1371/journal.pgph.0005146

**Published:** 2025-11-03

**Authors:** Claire Jacqueline Calderwood, Edson Tawanda Marambire, Modester Ngwerume, Maureen Tshuma, Mikaela Coleman, Trevor Musunzuru, Sibusiwe Sibanda, Evelyn Muringi, Karlos Madziva, Tinashe Bhaudi, Fredrick Mbiba, Lovemore Chupa, Fungai Kavenga, Collins Timire, Junior Mutsvangwa, Rashida Abbas Ferrand, Katherine Fielding, Justin Dixon, Katharina Kranzer

**Affiliations:** 1 Department of Clinical Research, Faculty of Infectious & Tropical Diseases, London School of Hygiene & Tropical Medicine, London, United Kingdom; 2 The Health Research Unit Zimbabwe, Biomedical Research and Training Institute, Harare, Zimbabwe; 3 CIH^LMU^ Center for International Health, University Hospital, LMU Munich, Munich, Germany; 4 Bordeaux Population Health, The University of Bordeaux, Bordeaux, France; 5 National AIDS and TB Programme, Ministry of Health and Child Care, Harare, Zimbabwe; 6 Biomedical Research and Training Institute, Harare, Zimbabwe; 7 Department of Infectious Disease Epidemiology, Faculty of Epidemiology and Population Health, London School of Hygiene & Tropical Medicine, London, United Kingdom; 8 Department of Global Health and Development, Faculty of Epidemiology and Population Health, London School of Hygiene & Tropical Medicine, London, United Kingdom; 9 Division of Infectious Diseases and Tropical Medicine, University Hospital, LMU Munich, Munich, Germany; NYU Grossman School of Medicine: New York University School of Medicine, UNITED STATES OF AMERICA

## Abstract

Globally, tuberculosis incidence and mortality is driven by syndemic interactions of tuberculosis with other chronic conditions including HIV, diabetes and undernutrition in a deleterious social and structural context, often characterised by poverty. Systematic screening for tuberculosis among household contacts is a core element of the WHO tuberculosis strategy but is hampered in high-tuberculosis incidence settings by health system constraints and low participation by household members of people with tuberculosis. Reframing screening as a health check, informed by the syndemic framework, could improve uptake and address proximate determinants of tuberculosis. Within a larger research study aimed at evaluating new tuberculosis diagnostic tests we developed and, using mixed methods, evaluated an integrated health check in a prospective cohort of tuberculosis household contacts in Zimbabwe. This included screening for a range of health conditions, health education and counselling, and on-site treatment or referral. Of 836 identified household contacts, 700 (84%) participated in tuberculosis screening. Of those, 467 people (67% women, median age 28 years) were invited to the health check; all participated in the intervention. One percent (n = 5/459) were diagnosed with tuberculosis. Almost two thirds (n = 288) had at least one unmet health need (either undiagnosed or uncontrolled diabetes, hypertension, HIV, anaemia, undernutrition, common mental health disorders, vision impairment, or tuberculosis). Of those referred following the health check, 66% accessed care for at least one condition, with variation across conditions. In-depth interviews with participants (n = 28), informed development of a refined explanatory theory, illustrating the benefits of a syndemic theory-based approach to tuberculosis screening for household contacts. Members of tuberculosis affected households have multiple, intersecting and unmet health needs. A holistic approach to systematic screening of household contacts guided by the syndemic framework could improve the health of these vulnerable people, advancing progress towards both tuberculosis and sustainable development goals.

## Introduction

Syndemic theory describes how different diseases concentrate and interact in a context due to shared upstream drivers [1]. Tuberculosis is a component of multiple syndemics including those of tuberculosis-HIV [2], tuberculosis-diabetes [3], tuberculosis-undernutrition [4], and tuberculosis-depression [5,6]. Upstream drivers of tuberculosis include structural (poverty, food insecurity, marginalisation) and community or individual level factors (poor healthcare access, stigma and discrimination, smoking, alcohol, and substance misuse) [7].

**Fig 1:**
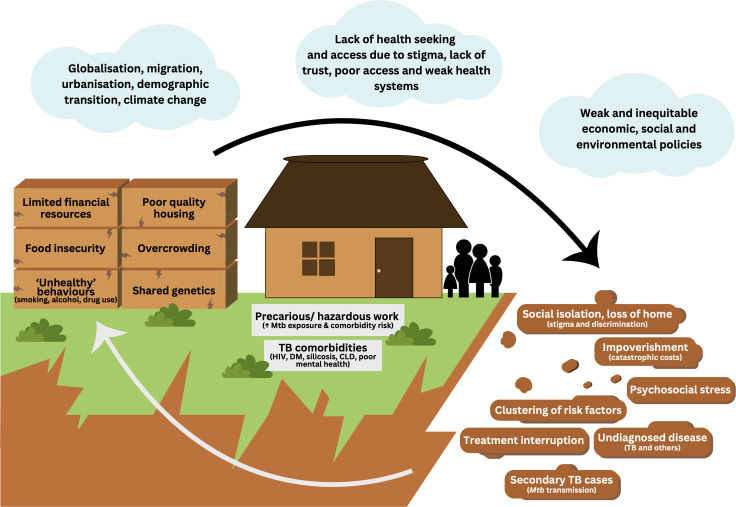
The household as the ‘unit’ by which tuberculosis is experienced, and thus unit of potential intervention. Footnotes: Tuberculosis most affects families exposed to adverse upstream determinants of health (illustrated with ‘clouds’; from [7]), which results in difficult life circumstances, and clustering of proximate determinants of tuberculosis incidence and poor health outcomes in households (illustrated with orange ‘bricks’). For individuals, these household-level factors drive tuberculosis exposure (through precarious/hazardous work and other pathways) and the development of chronic conditions including those associated with tuberculosis (tuberculosis comorbidities). Tuberculosis in turn has multiple consequences for families (illustrated with brown ‘rockfall’), driving a vicious cycle of recurrent tuberculosis episodes, poor health and increasing poverty. Abbreviations: *Mtb* = Mycobacterium tuberculosis, TB = tuberculosis.

Descriptions of tuberculosis-related syndemics have generally focussed on vulnerabilities experienced by the individual. There are strong arguments for shifting towards a family-centred approach: families share biology and behaviours and are subject to the same or similar drivers of poor health (Fig 1) [8]. Previous studies have described the fragile livelihoods and high prevalence of chronic conditions and associated risk factor clusters [9–12] among members of tuberculosis-affected households, with evidence of an excess risk of chronic conditions among members of tuberculosis-affected households compared to community controls [13–15]. Further, the psychological burden of tuberculosis on family members and need for psychosocial support is well documented [16–19]. These data support the development of coordinated ‘syndemic sensitive’ interventions, such as syndemic care [20]. Whilst integrated approaches to mass, community-wide screening are gaining traction, very few studies have sought to deliver such interventions in the context of tuberculosis household contact screening. The ZAMSTAR study in Zambia and South Africa included a household-based tuberculosis and HIV education, counselling and screening intervention for households affected by tuberculosis, received by 6.1% of the community [21]. Despite this small community reach, an endline tuberculosis prevalence survey suggested lower tuberculosis prevalence in intervention compared to control communities (adjusted risk ratio 0.82, 95% confidence interval 0.64–1.04), but the difference was not statistically significant. Another study in Myanmar found combined tuberculosis/non-communicable disease (NCD) screening for tuberculosis-affected households achieved high coverage and yield [13]. Treating an occurrence of tuberculosis as a sentinel of broader disease vulnerability could identify families who will derive the greatest benefit from interventions to improve health and wellbeing.

Systematic screening for tuberculosis and provision of tuberculosis preventive therapy (TPT) to tuberculosis-affected household members (i.e., household contacts) is a core element of the WHO End TB strategy, aiming to diagnose tuberculosis earlier and prevent onward transmission [22]. However, implementation of screening is often incomplete [6]. Further, when screening is offered, many household contacts do not participate due to barriers including low trust in services, negative anticipated consequences of diagnosis, paired with lack of perceived benefit, and other competing demands [23]. No studies have sought to explore the perspectives of TB household contacts on integrated approaches to screening and care, linkage to care after screening or the impact of this approach on non-TB specific health metrics. In one study in South Africa (where screening was for tuberculosis and HIV), inclusion of other chronic disease screening within tuberculosis contact tracing was suggested by community members as a means to increase the value of screening [24].

We aimed to determine the potential of a ‘health check’ integrated within systematic screening for tuberculosis among household members. Our approach was premised on the hypothesis that this holistic approach to screening, informed by the syndemic framework, would advance progress toward global tuberculosis goals, whilst improving health and wellbeing with a specific focus on the most vulnerable (a central premise of the Sustainable Development Goals). Specifically, we determined acceptability and yield of integrated screening for tuberculosis, HIV, diabetes, hypertension, anaemia, underweight, mental health and vision impairment in Harare, Zimbabwe, and assessed health-related quality of life and disease control status 12 months later. We used realist evaluation-informed thematic analysis [25] to understand whether and how the intervention worked through examination of context, mechanisms and outcomes.

## Methods

### Governance, ethics and reporting standards

This was a mixed-methods prospective cohort study with a sequential explanatory design. It included an intervention (health screening) at baseline and 12-month period of follow up; as well as in-depth participant interviews which were informed by the quantitative data. Informed written consent was obtained from all participants aged at least 18 years; for younger participants individual assent and written guardian consent by a was obtained. Ethical approval was granted by the ethics committee of the Medical Research Council Zimbabwe (MRCZ/A/2618), and the London School of Hygiene & Tropical Medicine, United Kingdom (22522–2). The study sample size was determined by the primary aims of the parent study (namely, to determine the diagnostic accuracy of novel tests for tuberculosis [26]). A sample size calculation was performed in advance to demonstrate adequacy of the sample size for this sub-study (Table A in S1 Appendix). The study was developed, implemented and analysed with involvement of local researchers, policymakers and local community, ensuring contextual relevance. The study has supported capacity development, including formal training opportunities. The intervention is described using the template for intervention description and replication (TIDieR) checklist (Table B in S1 Appendix), and qualitative analysis in accordance with the consolidated criteria for reporting qualitative research (COREQ) standards (Table C in S1 Appendix); additional methodological details are provided in S1 Appendix.

### Intervention development

We have previously described the current evidence base, rationale and Zimbabwean context for integrating a health check in screening of tuberculosis household contacts [8]. The specific intervention was developed by the research team, building on our previous experience of similar interventions in Zimbabwe [27], and in collaboration with a community advisory board. We included conditions which i) had known biological associations with tuberculosis, ii) were of public health importance in Zimbabwe (i.e., highly prevalent), or iii) were valued by community members (Table D–E in S1 Appendix). We required that the condition was measurable using a point-of-care tool in routine use and for an appropriate place for onward referral and treatment to be available in the local setting.

The initial programme theory (Fig A in S1 Appendix) was summarised as: if people from tuberculosis-affected households are offered additional services, then they will be more willing to participate in tuberculosis screening because of greater perceived benefit, and will be diagnosed with important conditions earlier, enabling them to access treatment, and resulting in improved quality of life.

### Study context and population

This study was nested within the Zimbabwe site of ERASE-TB, a multi-country cohort of tuberculosis household contacts aimed at evaluating tests to diagnose tuberculosis earlier [26]. The tuberculosis incidence in Zimbabwe is 211/100,000 population (2023) [6]. In Harare, the capital city where this study was based, most people with tuberculosis are residents of high-density, socio-economically deprived, urban neighbourhoods (unpublished, local notification data). Adults with tuberculosis were identified through all local primary care clinics in Harare and, following informed consent, asked to provide details of their household members. All household members aged 10 years and older of the enrolled adults with tuberculosis were then invited (in person or by phone) to participate in regular tuberculosis screening (comprising a World Health Organization tuberculosis four-symptom screen and chest radiograph, followed by Xpert Mtb/Rif Ultra [Cepheid] if either suggested tuberculosis) every six months for up to two years as part of ERASE-TB. Weekend and holiday visits sought to maximise uptake and retention, particularly among people in work. For this nested sub-study, all household members participating in ERASE-TB were invited to participate in an integrated health check (20/04/2022 – 27/03/2023) at the same time, and at the same location, as their baseline study visit or, as this sub-study started after that of the main cohort, at the six- or 12-month screening visit (Table F in S1 Appendix). The health check was delivered by a nurse and research assistant from tents at a central hospital (to accommodate COVID-19 infection prevention and control precautions). To facilitate accessibility (particularly in the context of COVID-19 restrictions [28]) at the central site, we provided transport.

### Intervention procedures

All participants were offered one-off screening for tuberculosis, HIV, under/overweight, anaemia and distance vision impairment, as described in Table C in S1 Appendix; all testing was point-of-care with results available within the study visit. Participants aged 14 years and older were offered an audio computer-assisted self-interview (ACASI) Shona Symptom Questionnaire (a locally developed and validated 14-item screening tool for common mental health disorders [29]; no screening was performed for participants <14 years due to lack of a suitable screening tool); participants aged 18 and older were offered screening for hypertension and diabetes (point-of care HbA1c); those 40 years and older were, in addition, offered screening for near vision impairment (Peek Vision tests). Participants were able to verbally opt in or out of each screening procedure. A positive screening result was defined using internationally agreed definitions (Table E in S1 Appendix). Health screening procedures were conducted by research nurses, trained in specific procedures and guided by a tablet application. Whilst we use the terms diabetes and hypertension, these are based on screening on a single day; we acknowledge that this approach does not fulfil diagnostic criteria [30,31].

Participants screening positive for any condition and those with a previous diagnosis but uncontrolled or untreated disease either received an on-site intervention or were referred for further assessment and care, facilitated by a nurse from the study team (Table E in S1 Appendix). For all conditions we intended for the initial point of access to care to be free of cost.

### Data collection

Quantitative data collected at baseline included an electronic research questionnaire capturing demographic details, pre-existing medical conditions, health behaviours (e.g., smoking, alcohol and substance use), barriers to healthcare access, and health related quality of life (using EQ5D-5L for adults and EQ5D-Y for adolescents <16 years). Participation in and results of screening tests were extracted from tablet-based screening tools. Methods for ascertaining linkage to care varied by condition and included direct reporting from providers, telephone contact with participants 8–12 weeks after referral, and a questionnaire at 12 month follow up.

Participants were followed up in person 12 months after participation in screening. Disease ‘control’ was assessed for diabetes, hypertension, anaemia, and mental health by repeating tests used for screening (Table E in S1 Appendix). Participants with vision impairment were asked whether they had glasses and wore these regularly. HIV viral load testing was performed for participants with HIV, either immediately, with feedback of results, for those who wished to have a viral load test, or anonymously on stored samples for those who did not. Health-related quality of life was again measured using EQ5D tools [32]. People who reported that they were living with a health condition at follow up were asked to complete an interviewer-administered structured questionnaire about time and costs associated with accessing healthcare (adapted from the tuberculosis patient cost survey tool [33]). Whilst we collected data on incident TB diagnoses and occurring during the follow up period and TB treatment outcome data, these will be reported in a separate manuscript.

A purposively selected subset of participants to reflect a range of ages and ensure gender balance, including people who had/had not screened positive for a condition and those who had/had not linked to care, were invited to participate in semi-structured in-depth interviews (Table G in S1 Appendix). Interviews were conducted in Shona or English by MT and MN, according to participant preference, parallel to delivery of the health check (either at the time of the health check and during follow up). Each participant was interviewed once. The initially developed topic guide was iteratively refined during data collection in response to both quantitative and qualitative data (S1 Appendix). Interviews were audio-recorded, transcribed and translated into English where required. The final sample size was guided by the principle of saturation in thematic analysis [25].

The intervention was delivered by members of a research team, all of whom are authors. The research team’s perspective was captured during intervention development and delivery through regular, documented, team meetings, field notes (CJC, BS, MN), and a group discussion of the findings. We additionally kept detailed minutes of community advisory board meetings throughout intervention development and delivery.

### Quantitative data analysis

We report uptake, defined as the proportion of identified household members who participated in tuberculosis screening, and, separately, the proportion of those invited who participated in the integrated health check. Yield was defined as the proportion of screened individuals who screened positive for at least one and for each screened condition. Proportion linked to care was the proportion of people referred who had at least one contact with a health provider regarding that condition. We assessed medium term outcomes: the proportion of participants who had controlled disease (as defined in Table E in S1 Appendix) at 12 month follow up, and the difference in mental health symptoms and health-related quality of life between baseline and follow up.

We explored concentration and interactions between diseases by graphically displaying overlapping conditions at an individual and household levels (Euler plots), describing the most common disease dyads and triads (multimorbidity) and calculating correlations between pairs of conditions at an individual level.

### Qualitative data analysis

Interview transcripts and field notes were imported into NVivo for analysis. The overall aim of realist evaluation is to identify the underlying generative causal mechanisms that explain how outcomes were caused and how these are influenced by context [34]. We selected this approach over considered alternatives in response to initial review of the data, which revealed that participant experiences of the intervention were deeply embedded in context. In the analysis, we thus wished to centre emergent mechanisms and outcomes in their specific context. Thematic analysis was conducted (CJC, MN, MT) using a grounded theory approach, mapping emergent themes (Table H in S1 Appendix) to the core realist evaluation domains of context, mechanisms and outcomes and searching for relationships between themes and domains. The research team discussed the data as a whole (triangulating quantitative and qualitative findings), through which a refined middle-range programme theory was developed.

## Results

In this section, the quantitative findings (uptake and yield of screening, and linkage to care) are reported first, followed by the qualitative findings, framed in terms of intervention context, mechanisms and outcomes.

### Uptake of screening

**Fig 2:**
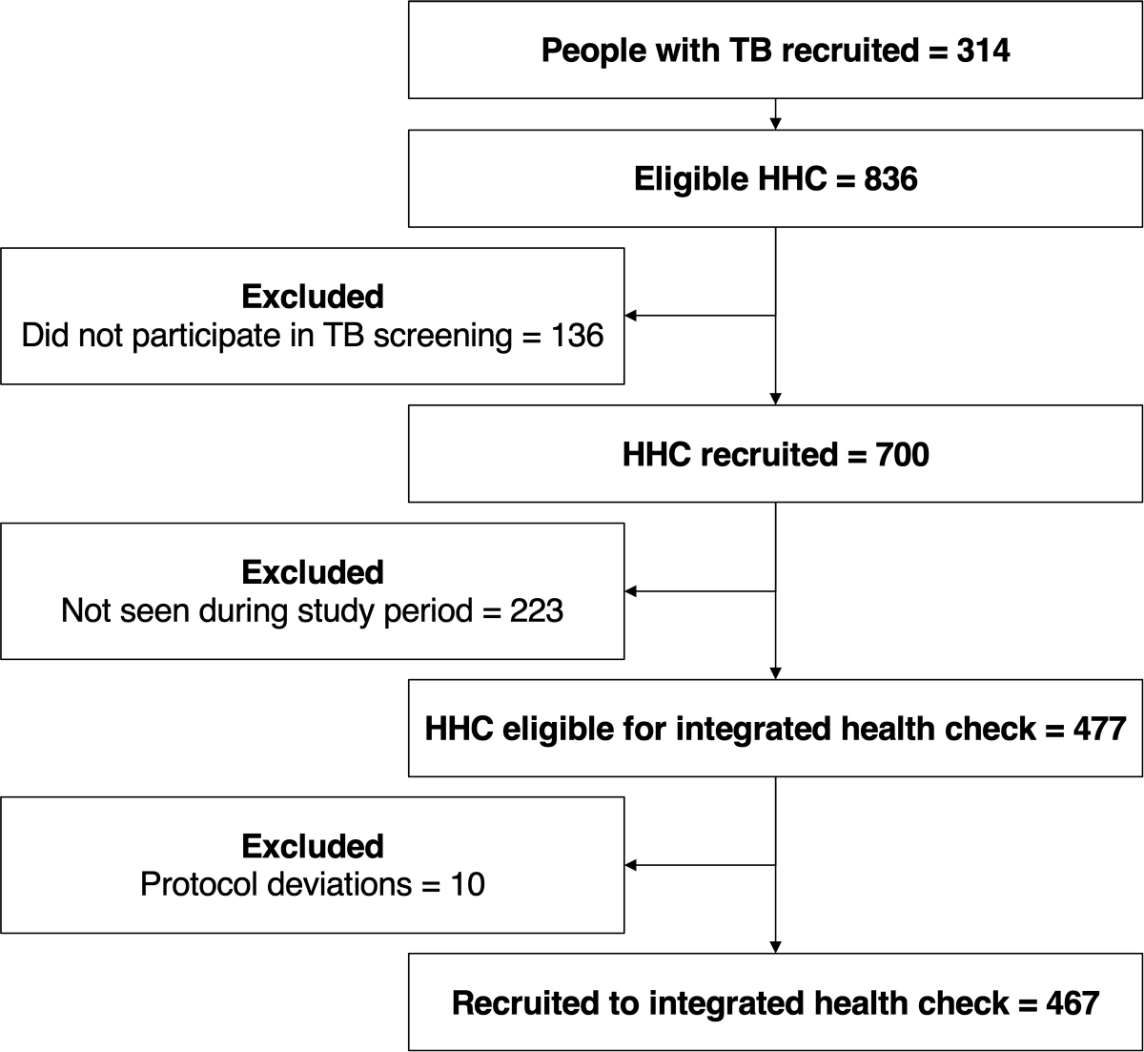
Flow diagram of study participation. Footnotes: Eligible household contacts (HHC) is the number of people reported by the person with TB as being over the age of 10 and living in their household.

Overall, 314 people with tuberculosis reported having household members aged over 10 years and consented to these being notified about their tuberculosis status; from these 836 eligible household members were identified and 700 (84%) participated in the tuberculosis screening study. Of these, 223/700 were recruited outside the enrolment window for the integrated health check, resulting in 477 eligible household members (n = 181 households). Ten people were erroneously not offered the health check as it was assumed they had already participated; all 467 invited household members participated (Fig 2).

Among participants, 64% were women and median age was 28 years (interquartile range 17–43 years; Table 1). Ninety one percent of participants aged under 18 years were currently enrolled in school. Of participants (all ages) not currently enrolled in school, 83% had completed secondary education and 37% were in formal employment. Nine percent of participants (20% of men) were current smokers and 4% (10% of men) had a history of work in the mining sector. Almost half (46%) of participants reported the cost of healthcare to be a usual barrier to access (Table I in S1 Appendix).

**Table 1:** Characteristics of members of tuberculosis-affected households participating in the health check.

Characteristic		Overall N = 467	Men N = 168	Women N = 299
**Demographics**				
Age, years		28 (17–43)	22 (15–40)	30 (19–43)
Age category	10-18 years	119 (25%)	55 (33%)	64 (21%)
	18-39 years	211 (45%)	69 (41%)	142 (47%)
	40 + years	137 (29%)	44 (26%)	93 (31%)
Currently enrolled in school (if <18 years)		108 (91%)	52 (95%)	56 (88%)
Highest education attained*	At least secondary	280 (83%)	96 (91%)	184 (79%)
	None or primary	58 (17%)	10 (9.4%)	48 (21%)
Employment status*	Formal	133 (37%)	66 (57%)	67 (28%)
	Informal/ occasional	40 (11%)	7 (6.1%)	33 (14%)
	Homemaker/ retired	33 (9.2%)	15 (13%)	18 (7.4%)
	Unemployed	151 (42%)	27 (23%)	124 (51%)
History of work in mining		19 (4.1%)	17 (10%)	2 (0.7%)
History of living in South Africa		66 (14%)	23 (14%)	43 (14%)
History of imprisonment		13 (2.8%)	12 (7.1%)	1 (0.3%)
Currently pregnant		21 (7.6%)	–	21 (7.6%)
Private medical insurance cover		40 (8.6%)	18 (11%)	22 (7.4%)
Physical activity (IPAQ-SF)	Low-Moderate	243 (52%)	72 (43%)	171 (57%)
	High	224 (48%)	96 (57%)	128 (43%)
**Health behaviours**				
Current smoker		40 (8.6%)	34 (20%)	6 (2.0%)
AUDIT category**	Non-drinker	276 (71%)	61 (48%)	215 (81%)
	Non-harmful drinking	73 (19%)	36 (29%)	37 (14%)
	Harmful drinking	18 (4.6%)	12 (9.5%)	6 (2.3%)
	Alcohol dependence	23 (5.9%)	17 (13%)	6 (2.3%)
Ever used recreational drugs^†^		50 (13%)	34 (27%)	16 (6.1%)
**Medical history**				
Previous tuberculosis		25 (5.4%)	12 (7.1%)	13 (4.3%)
Known HIV		65 (14%)	16 (9.5%)	49 (16%)
Known diabetes		10 (2.1%)	2 (1.2%)	8 (2.7%)
Known hypertension		60 (13%)	8 (4.8%)	52 (17%)
Known mental health condition		3 (0.7%)	1 (0.7%)	2 (0.7%)
Known visual impairment		39 (8.4%)	14 (8.3%)	25 (8.4%)
Known lung disease		38 (8.3%)	14 (8.4%)	24 (8.2%)
Ever previously broken a bone		30 (6.4%)	16 (9.6%)	14 (4.7%)
≥1 COVID-19 vaccine received^‡^		254 (58%)	84 (54%)	170 (60%)
**TB prevention**				
Received TPT (last 12 months) ^§^		11 (2.4%)	2 (1.2%)	9 (3.0%)
BCG vaccinated		234 (98%)	84 (97%)	150 (98%)
BMI (kg/m2)		22.2 (19.2–26.6)	19.6 (17.9–21.9)	24.2 (20.9–28.4)
**Household-level measures**		**N = 181** ^¶^		
Income <2.15USD per person per day^||^		148 (87%)		
Income in USD		0.82 (0.41–1.64)		
Household crowding		48 (27%)		
Moderate-severe food insecurity (FIES)		60 (34%)		
Primary income earner has tuberculosis		86 (48%)		

**Footnotes**: estimates presented as median (25%–75%) or n (%). School enrolment calculated among participants aged <18 years only. * Highest education and employment status calculated among people not currently enrolled in school; education missing for 12 people and employment status missing for 2 people not currently enrolled in school. ** AUDIT categories were defined as non-drinker (score 0), non-harmful drinking (score 1–7), harmful drinking (score 8–14) and alcohol dependence (score 15+). † Use of substances assessed using the alcohol, smoking and substance involvement screening test (ASSIST) among participants aged ≥14 years (n = 406) and was additionally missing for n = 23. Of 50 people who reported using recreational drugs, 24 used cannabis, 6 methamphetamines and 3 cocaine, the remainder did not disclose. ‡ 26 people aged <12 years not included as not eligible for COVID-19 vaccination. § Among people currently taking or who had taken TPT in the past 12 months 9 (81.8%) were living with HIV. ¶ N reported is the number of households represented by the study population. Household income was missing for 11 households; food security was missing for 4 households. ^||^ 2.15USD per person per day corresponds to the poverty line used in national reporting. For context, 39.8% of Zimbabwean nationally and 11% urban residents had a household income of less than 2.15USD per person per day in 2019 (most recently available data). †† Household crowding was defined as ≥3 people per habitable room, as per UN Habitat [35]. **Abbreviations**: AUDIT = Alcohol use disorders identification test; BMI = Body mass index; IPAQ-SF = International physical activity questionnaire - short form; FIES = Food insecurity experience scale; N = number; TPT = Tuberculosis preventive therapy; USD = United States Dollars.

**Fig 3:**
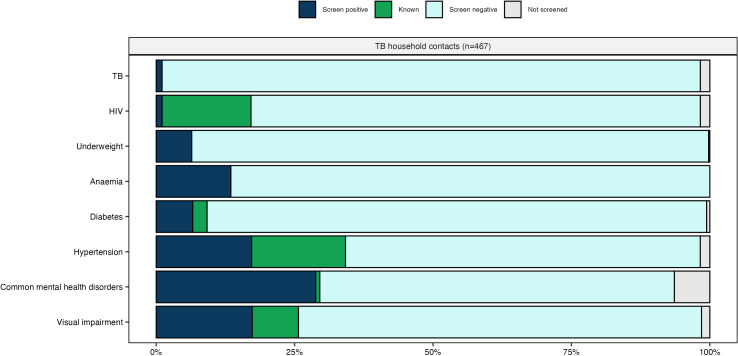
Uptake and yield of screening for chronic conditions among members of tuberculosis-affected households. Footnotes: Screening for diabetes and hypertension was not indicated among people aged <18 years (n = 119 [25.5%]) and screening for common mental health disorders was not performed among people <14 years (n = 61 [13.1%]). Individuals in which screening was not indicated are excluded from the Fig.

Among people who participated in the health-check, uptake of all individual components was very high with over 95% of eligible people completing each component other than mental health assessment (6.4% declined; Fig 3 and Table J in S1 Appendix).

### Yield of screening

**Fig 4:**
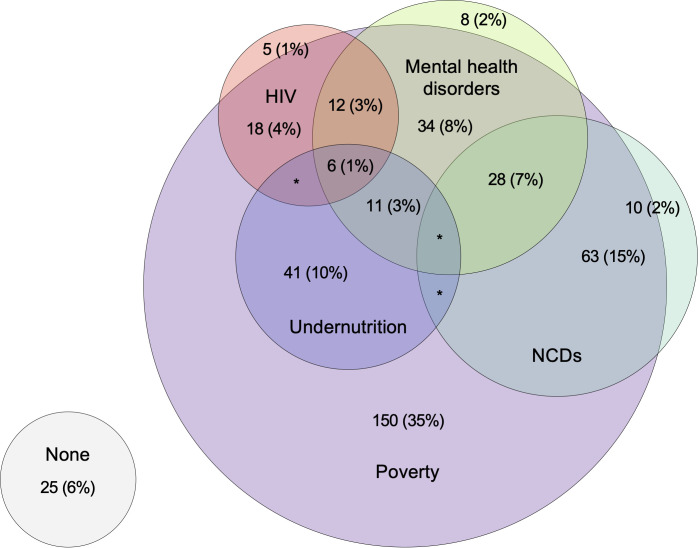
Overlap of communicable, nutritional, mental and non-communicable diseases among members of TB-affected households. Footnotes: Numbers (percentages) presented. Poverty defined as household income less than 2.15 United States Dollars per person per day and defined at a household level. All other variables defined at individual level. Nutritional needs defined as underweight or anaemia (defined using World Health Organization thresholds), NCDs as diabetes or hypertension (either known or detected at screening). Areas indicated * contain 5 or fewer individuals (<1%).

Overall, 66% (n = 310) of participants had at least one undiagnosed or uncontrolled chronic condition identified by the health check, with 55% (n = 257) screening positive for at least one health problem (Table K in S1 Appendix). One percent (n = 5/459) of participants were diagnosed with tuberculosis (overall 5.4% [n = 25] had previous tuberculosis), 1.5% (n = 7/452) were newly diagnosed with HIV (overall HIV prevalence 17%), 6.6% (n = 23/346) screened positive for diabetes (overall prevalence 9.2%), 17.5% (n = 60/342) screened positive for hypertension (overall prevalence 35%), 31% (n = 117/380) screened positive for common mental health disorders (overall prevalence 32%) and 18% (n = 81/460) screened positive for vision impairment (overall prevalence 26%). A total of 6.4% of participants were underweight (n = 30/466) and 14% (n = 63/467) were anaemic; none of these were previously identified. Thirty five percent of people had multimorbidity (defined as two or more of HIV, diabetes, hypertension, common mental health disorders, vision impairment, underweight or anaemia), increasing at older age. There was substantial overlap between communicable, nutritional, mental and NCDs, superimposed on financial insecurity (Fig 4 & Figs B–C in S1 Appendix).

### Linkage to care

All 243 people meeting criteria for referral were referred. Of those, 161 (66%) accessed care for at least one condition. The proportion of participants who had accessed care at least once following screening varied across conditions: the highest proportion of people accessing care were those with a diagnosis of tuberculosis (n = 5/5) or HIV (n = 8/9) and lowest were those screening positive for diabetes or hypertension (n = 9/31 and 41/97 respectively; Table 2). People who were older were more likely to link to care for diabetes or hypertension (Table L–M in S1 Appendix). For mental health, vision impairment and underweight, half to four-fifths of people linked to care (n = 75/120, 42/74 and 25/30 respectively); importantly, these interventions (unlike those for HIV, diabetes and hypertension) were delivered by non-governmental organisations or the study team (food hampers delivered to households).

**Table 2:** Referrals, linkage to care and disease control at 12 months among members of tuberculosis-affected households.

Condition	Baseline (N = 467)		Referral	Follow up (N = 398)
	Prevalence	% Controlled*	Linked to care†	% Controlled*
HIV	79/458 (17.2%)	70/79 (88.6%)	8/9 (88.9%)	44/53 (83%)
Underweight	30/466 (6.4%)	0/30 (0%)	25/30 (83.3%)	6/30 (20%)
Anaemia	63/467 (13.5%)	0/63 (0%)	0/2 (0%)	41/56 (73.2%)
Diabetes	37/463 (8%)	6/37 (16.2%)	9/31 (29%)	29/32 (90.6%)
Hypertension	119/458 (26%)	22/119 (18.5%)	41/97 (42.3%)	36/92 (39.1%)
Common mental health disorders	120/380 (31.6%)	0/120 (0%)	75/120 (62.5%)	68/101 (67.3%)
Vision impairment	120/460 (26.1%)	6/120 (5%)	42/74 (56.8%)	36/101 (35.6%)

**Footnotes**: * Number and percentage controlled at each timepoint; denominator is those with the condition at baseline, at follow up this is restricted to those participants with follow up complete. Definitions of disease control/uncontrol at baseline and 12 months (m) are in S1 Appendix. For HIV, only people with known viral load were included in the denominator. 65 people with HIV were followed up, of which 5 did not have a viral load performed and invalid results were returned for 7. † Number linked to care, and as a proportion of all those referred. Linkage to care was defined as having at least one encounter with a health professional about the condition (as defined in the S1 Appendix). For anaemia, only participants with severe anaemia (Hb < 8g/dL) were referred, the remainder were given dietary advice. For vision impairment, participants with distance vision impairment were referred, those with near vision impairment (>40 years only screened) were given reading glasses at the health check visit (81% with near vision impairment received glasses). For all other conditions, all people with undiagnosed or uncontrolled disease were referred.

Follow up was completed for 398 participants (85%; median 376 days [IQR 331–506 days] from screening; completion of follow up 24/05/2024). Three participants (0.6%) had died in the intervening period (one possibly due to TB), two opted out of the study and the remainder (n = 64) were lost to follow up. Fewer participants had symptoms of common mental health disorder at follow up compared to baseline (19% vs 32%, p < 0.001) whilst EQ5D values were similar (p = 0.09; Table 3). In explorative analyses, the difference in Shona Symptom Questionnaire [29] scores between baseline and follow up appeared to be driven by large reductions in the proportion of participants reporting symptoms which may reflect more acute, reactive changes in mood (Table N in S1 Appendix). There was no difference in mental health scores or EQ5D among people who did and did not access care after screening (Table O in S1 Appendix). The proportion of participants with chronic conditions at baseline who were classified as having disease ‘control’ at follow up varied considerably by condition, with the highest proportion for people with diabetes (91%) and the lowest for those who were underweight (20%; Table 2). Participants spent a median of 28 (IQR 11–97) United States Dollars each on assessing healthcare in the period between screening and follow up (Table P in S1 Appendix).

**Table 3:** Change in Shona Symptom Questionnaire scores and EQ5D values between baseline and follow up.

		Baseline	Follow up	P value
		N = 380	N = 360	
SSQ	Score	4 (2 –8 )	3 (1 –6 )	<0.001
	Positive screen	120 (31.6%)	67 (18.6%)	<0.001
		**N = 374**	**N = 330**	
EQ5D-5L value	Score	0.90 (0.85–0.90)	0.90 (0.85–0.90)	0.09
	No difficulties	231 (61.8%)	183 (58.7%)	<0.001

**Footnotes**: Presented as median (interquartile range) or number (percentage). Shona Symptom Questionnaire (SSQ) completed by all consenting participants aged ≥14 years and EQ5D-5L completed by all consenting participants aged ≥16 years; values calculated with crosswalk to the Zimbabwe EQ5D-3L value set. P values presented for paired t-tests or chi squared tests comparing baseline and follow up results.

### Intervention context, mechanisms and outcomes

**Fig 5:**
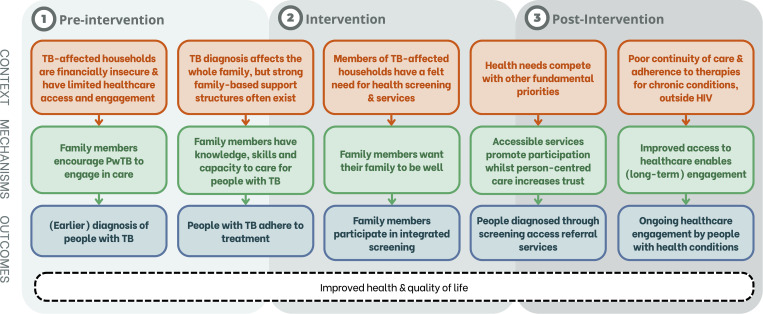
Refined programme theory of how an integrated health check for members of tuberculosis-affected households improves health and wellbeing. Footnotes: The longitudinal programme theory spans the period before household members are invited to participate in screening (pre-intervention), the intervention (including screening and linking to ongoing care) and the period of maintenance after the intervention (post-intervention). Abbreviations: PwTB = person/people with TB.

A total of 28 members of TB affected households (15 women, median age 35 years) were interviewed (Table D in S1 Appendix). Key themes are narratively summarised below and reported, with example quotes, in Table Q in S1 Appendix. Quotes are suffixed with participant dempgraphics (M = male and F = female) and whether they were diagnosed with any conditions during the screening intervention (DM = diabetes; HTN = hypertension; CMD = common mental health disorder). Fig 5 frames the findings of this study in a context-mechanism-outcome format, forming a refined intervention explanatory theory [36]. Questionnaire data on experiences of healthcare seeking are summarised in Table I & L in S1 Appendix).

1) *Tuberculosis-affected households are financially insecure and have limited healthcare access and engagement*

Zimbabwe has experienced a sustained economic crisis resulting in limited healthcare expenditure. Most funding comes from external agencies as disease-specific health programming, with the consequence being a fragmented, plural healthcare landscape [37]. For members of TB-affected households who were less likely to have medical insurance, more likely to be food insecure and had lower household incomes [38] compared to the average for urban areas in Zimbabwe, user fees at public sector facilities were cost-prohibitive, and medication stockouts were a common experience.

“It was after [the index case] was seriously sick [that] we went to the hospital because at first we were reluctant because of the expected bills, but then we got help from [a neighbour].” (34M, no diagnoses)

While provision of care was considered good, particularly for tuberculosis where medicines were usually available and patients were prioritised, healthcare workers could be perceived as impatient or unkind, felt to reflect their professional or personal stresses. These intersecting factors made accessing healthcare a challenge that, for interviewees, required “strength” to overcome.

“The way you are treated, it is very difficult in healthcare facilities. On some days the service may be good and on some days it can be bad. It depends on the person’s mood, some people bring their moods from home to the workplace.” (46F, HIV & HTN)

2) *Tuberculosis diagnosis affects the whole family, but strong family-based support structures often exist and facilitate care seeking and adherence*

Pre-existing chronic financial insecurity and psychosocial stress was multiplied by the tuberculosis episode that resulted in loss of income and increased expenditure. This meant that members of tuberculosis-affected households had urgent competing priorities (e.g., providing for the family).

“I had to sell most of the things that I had [when I developed TB]. I had to sell my car. I am the breadwinner so at the time that I fell sick, I needed medical attention, and I could no longer go to work. The only thing I could sell was my car so the children could go to school, and I could get medical attention.” (38M, no diagnoses)

Whilst in some households, disruption was temporary, for others, due to death or permanent disability, income restoration was at most partial. Strong family structures reaching beyond the household and often including extended family members created a support network. The multifaced support provided to the person with tuberculosis included psychosocial support, personal care, loans or gifts, whilst family members actuated healthcare seeking and sustained engagement.

“[My husband] started to have cough, and sweating at night and losing weight. And I would advise him to go and get tested but you know how it is with men… we would argue about going to the clinic but then he would just go and buy pills. Until I called my mother in law, she is the one that then told him to go and get tested.” (26F, HIV)

“I got help from my brothers. They helped me [with money and groceries] until [my husband] recovered.” (42F, HIV)

Most interviewees spoke about having a strong family unit that accepted the person with tuberculosis and adapted their roles to provide support, despite limited financial capacity and knowledge with which to do so. Lack of reliable information about tuberculosis caused family members to question why someone had developed tuberculosis – with possible reasons including alcohol, dust exposure, HIV or witchcraft. Many people believed tuberculosis to be fatal; this contributed to pervasive fear of infection (particularly among adolescents), fear of death, or fear of not providing.

“I did not know much about TB at that time and I still know very little about it. I thought that were was no difference between one who has TB and another who has AIDS.” (21M, no diagnoses)

“I knew that one can contract TB from dust and that people who work in mines or smoke are at high risk. Most people say that it is a very painful illness and it is rare for a person to survive after being diagnosed with TB.” (15F, no diagnoses)

The support most people with tuberculosis received from family member was contrasted with the attitude of “others” – usually other members of the community who excluded people directly or indirectly affected by tuberculosis. This sometimes resulted in eviction of families from their homes. In a minority of families stigmatising attitudes and “blame”, often arising from the association of tuberculosis with HIV, existed within the family, isolating the tuberculosis carer or carers.

“There was no one [who helped me]. People say different things…. They say many things, such as if someone is diagnosed with TB it means they are HIV positive. This makes life difficult. To continue living at the house you were living before becomes impossible. You will be forced to look for another house because the situation will not be good. People will not be comfortable around you and they will distance themselves.” (39F, HIV & CMD)

3) *Accessible, integrated services meet a felt need for health screening and services,* *increasing anticipated benefit and promoting participation*

Domination of HIV within the public health landscape of Zimbabwe shaped how members of tuberculosis-affected households understood their own health and how healthcare was experienced. Screening programmes were a familiar concept, with the rationale for participating (regardless of the disease being tested for) being to “know your status” or, in Shona, “*ziva paumire* [know where you stand]” – directly reflecting the language of HIV awareness campaigns. This familiar concept, paired with knowledge that tuberculosis is transmissible, meant screening was perceived as beneficial. However interviewees felt inadequate access to information about tuberculosis and NCDs might lead some people to not participate.

“It is good because we could be infected [with TB] and not know about it. So if we have regular tests, we will know where we stand.” (15F, no diagnoses)

High anticipated benefit of the intervention was facilitated by the opportunity of better treatment outcomes if someone had tuberculosis through earlier diagnosis, whilst a tuberculosis-negative result was seen as providing reassurance. The opportunity to know about other health conditions for which testing was not normally available or accessible was considered advantageous, providing an avenue towards treatment and the incentive to implement lifestyle changes to improve future health. Integration of screening for multiple different conditions both reduced the work associated with accessing testing and resulted in a sense of care for the whole person.

“Blood sugar, HIV, BP, and many others… eyesight… I was tested for mental health… I was actually very happy because I was tested for other diseases I have never been tested for… I like knowing my health status… I learned that I am fit and do not have any ailments. It is better than to live with suspicion.” (21M, no diagnoses)

For some, however, new NCD diagnoses were a source of stress when paired with limited contextual capacity to act.

‘I was so worried but I was happy for the programme… I was hurt because I thought I would become blind yet I have kids that need to be taken care of… I am worried that with time I might yet go blind.” (47F, vision impairment)

4) *In a context of limited reliable information about tuberculosis, education and* *counselling empower household members to provide care for people with tuberculosis and seek care in future*

Another highly valued dimension of the intervention was an increased capacity to support the person with tuberculosis. In the context of limited knowledge, social isolation and financial insecurity, participants expressed gratitude for the information and psychosocial support that the team provided, which had enabled them to care for the person with tuberculosis and allowed them to participate in screening through alleviating fear.

“What helped me was the counselling we received. Now I know that TB is treatable… Now I am able to counsel other people who come and ask about my husband’s sickness. If they want to know the process, I tell them and I offer counselling because I know that TB is treatable – as long as you follow the regimen and complete the course, you will live.” (39F, HIV & CMD)

The approach taken by the team and the environment in which the service was delivered also contributed to a sense of holistic care. Staff were seen as compassionate, taking time with each client, whilst also being well-trained and professional. Consequently, participants felt respected and valued, an experience which some contrasted with their previous healthcare encounters.

“The way you are handled speaks volumes on your health and even your mind… because at some of our regular hospitals, we might have given up way back because of how they speak to patients… [At the study] they always speak in a nice way without any harsh tones. They are good people and they speak with you until you understand and they do not force anything on you.” (40F, HTN, asthma, vision impairment)

5) *Push and pull factors influence engagement with screening and healthcare generally, including competing with other urgent and fundamental priorities, and stigma*

Person-centred care was facilitated by the services being free, and clients supported with transport to the screening site (based at a local hospital), making the intervention desirable. More convenient sites suggested (particularly if support for transport was not provided) included local clinics and community centres, with word of mouth and visibility of the programme within the community seen to be important in driving participation. No interviewees suggested home-based screening, but specific outreach to target marginalised groups (such as young people who use drugs) was suggested.

“[The programme] is good because it reduces the burden on those that do not have adequate financial resources. Nowadays it is cash up front. If you have a headache, cash first, in order to get assistance.” (34M, no diagnoses)

For some groups (people in school or formal employment) time conflicts were a barrier to accessibility, whilst those in informal employment weighed up the anticipated benefit of participating against the likely loss of income. The high anticipated benefit of the intervention meant most participants prioritised this over other commitments, however a few did not.

“There is a girl [in the house] who is 13 years old now, she never got tested… her and my husband never got tested because the times were clashing, because of work and school” (40F, HTN, asthma, vision impairment)

The potential for community gossip and discrimination was a deterrent to participating in screening or attending healthcare generally. For example, some participants asked to be met by the study team some distance from their homes and use of branded cars was considered unacceptable.

“I think [sometimes people] fear what others in the community think. Some people live in communities where people discuss each other’s lives daily. They will be afraid that people will be gossiping in the community, or start avoiding them.” (15F, no diagnoses)

Further, two participants questioned why support was only offered for health problems and highlighted the need for financial support, asking for the study to help with other needs such as school fees. In the context of the severe financial insecurity, this reiterated how the most fundamental and pressing needs for many people were not those related to their health.

“[The screening will have a bigger impact] by assisting the unemployed people to get jobs, to give medicines to those in need, to rehabilitate those who are going astray… You should give us jobs.” (43M, previous TB)

“Do you not have others that can help with the other things… like school fees, as for me, I want my kids to have a good education, but right now, they do not have.” (47F, vision impairment)

6) *Education, counselling and support provides motivation and ability to prioritise health whilst improved access to healthcare enables engagement*

Understanding of NCDs was limited. Several interviewees attributed their diagnosis to the psychosocial consequences of the recent household tuberculosis diagnosis, often through biologically plausible mechanisms (e.g., stress leading to high blood pressure and worse mental health), leading them to conclude that it would self-resolve in time.

“I used to work in a mine, we always worked with torches and at times slept with them on so I quickly understood that my eyes might have been a problem… I thought to myself, maybe my current situation is causing my high BP. I look after sick people at home and there is no way the mind will be calm in such a situation.” (42M, HTN & vision impairment)

Participants were also fearful about starting treatment for NCDs, including fear of side effects they had seen others experience during anti-retroviral therapy (despite these being different medicines), as well as the implications of starting treatment and subsequently stopping at some point in future (for example, because of lack of funds). This latter concern may reflect the context of intensive messaging around HIV drug resistance and the importance of complete adherence. It may also reflect misunderstanding about complications such as stroke being directly caused by stopping hypertensive treatment rather than uncontrolled disease. Several interviewees with hypertension mentioned the drugs being something for “later” when their illness became more severe, and a preference to use “herbs” in the first instance. This sentiment resonated with our community advisory board, who recalled similar fears in personal or professional experiences of HIV, where, particularly early in public awareness of the disease, people tried multiple alternative strategies to delay the start of medications.

“I would be lying if I spoke of medication. They only told me that my BP is high and I should go on treatment. I did not [go to the clinic]… I am afraid of taking medication.” (39F, HTN)

Overall, in contrast to HIV, understanding of the causes, complications and treatment of NCDs was limited, however understanding improved through participating in the health check and participants made changes to their diet and behaviours as a result.

“I reduced my salt intake and [they said] that I should exercise regularly.” (42M, HTN & vision impairment)

“I was told to stop taking heavy foods (carbohydrates) and fizzy drinks, especially Pepsi…. She also said I must eat sadza in small portions. I am still doing that to this day.” (39F, HTN)

Participants felt that it was important for screening to be combined with assistance to access ongoing care. Those who participated in the linkage to care process in this study (i.e., attended an appointment and/or received treatment) felt this worked well. However, multiple factors contributed to whether participants screening positive for chronic conditions accessed subsequent care. These related to perceptions and understanding of the condition, the referral pathway, the likely chronicity of treatment, and anticipated consequences of the disease or its treatment. For example, participants who accessed care for vision impairment felt positively about a (free-of-cost) one-off encounter, whereas those attending hypertension clinics sometimes later stopped attending due to the ongoing cost of the medications. For some participants, the path from screening to long-term treatment was complicated by ongoing hurdles including the repeated costs of transport (often due to inconvenient, secondary care-based facilities for NCDs) and medication, and medication stock outs.

“I use public transport [to collect my medication]. It is 50c one way… I cannot walk the distance because my chest feels heavy. I will not manage it.” (39F, HIV & CMD)

“There may come a time when I wake up and I am no longer able to work. That means I will no longer be able to purchase the medication… [My relatives] do not have money. They actually need assistance from me. If I fall sick then all will be lost…” (40F, HTN)

With the study community advisory board, we questioned whether our linkage to care procedures could be improved or have been better supported: they felt the level of support was appropriate but wider structural and organisational change (such as the need to attend secondary care to be initiated on hypertension and diabetes treatment), as well as improved community understanding about NCDs, were needed.

## Discussion

This study demonstrated a high burden of intersecting health needs among members of tuberculosis-affected households and, for the first time, outlines how an integrated health check approach to systematic screening could improve the health of vulnerable families affected by tuberculosis. From a participant perspective, this approach was desirable and delivered benefit – not only through identification and facilitation of treatment of chronic conditions but also through empowerment of household members to better support the person with tuberculosis, whilst increasing trust.

Eighty-four percent of identified household contacts participated in the tuberculosis screening intervention; none of those declined the add on integrated health check. This suggests that integrated health screening was highly acceptable among the population participating in tuberculosis screening. This study is among the first to offer integrated services to members of tuberculosis-affected households as part of systematic screening for TB, and the first to combine communicable (HIV) and non-communicable diseases in the same intervention [13,21,39]. Our data adds to the growing body of evidence that integrated approaches to conventionally vertical disease screening in low- and middle-income countries are both clinically and cost-effective [40,41]. Tuberculosis related stigma, including associative HIV-related stigma, remains an issue in many settings, including Zimbabwe, and is an important concern for tuberculosis household contact screening interventions. Here the integrated health check was only offered to people already participating in TB screening and thus we were not able to evaluate the impact of this approach on stigma influencing participation in screening by tuberculosis-affected households.

Low rates of linkage to care are common across many conditions and interventions in Africa, particularly relating to NCDs [40,42,43]. The proportion of people linking to care for NCDs in this study is similar to that reported elsewhere, including from studies where, unlike here, direct ongoing support was provided to clinics and services and continuous medications were free. However, we did support initial access costs; linkage to care may be lower in a programmatic setting if such support is not provided. In our study, linkage to treatment for HIV and tuberculosis was almost complete – much higher than in some other southern African settings however numbers were relatively small [40,44–48]. Many previously-described health system barriers to long term engagement in NCD care were evident [49–51], however individuals were also reluctant to start medication due to other personal factors. There is an urgent need for greater availability of, and better, NCD messaging which highlights the importance of medication as prevention of future ill health.

An important finding of this study was the central role of family in supporting a person with TB, and how the family adapted for the loss of the key role this person played in family life. Participants tended to be more concerned about the wellbeing of others than themselves, shaping the mechanisms and perceived benefit of the intervention. This emphasis on family meant that our services were not viewed as person-centred, but rather family-centred. We did not anticipate direct benefits of the household member health check for people with tuberculosis, however, it was very clear from participant interviews that the education and counselling household members received improved their capacity to care for the person with tuberculosis. This is likely to have had impacts on outcomes for people with tuberculosis not measured here [23]. Other studies have described how not disclosing tuberculosis status to family or lack of family support is associated with not accessing tuberculosis treatment [52,53], non-adherence, adverse treatment outcomes [54,55] and worse wellbeing [56]; whilst patients with tuberculosis identify family support as being crucial to their recovery [16–18,57,58]. Family-centred care [59] and health services that draw from principles of *ubuntu* [60] have each been proposed as approaches to centre connectedness, respect, information-sharing and collaboration in health service delivery. With regard to families, *ubuntu* recognises the core role of family in wellbeing and forming a social safety net by providing logistical, financial and psychosocial support, particularly during difficult times [60]. This was an emergent theme here, perhaps surfacing why integrated, family-centred approaches to tuberculosis screening and care were acceptable and highly valued by community members in a Zimbabwean context. Simultaneously, the dissonance between current approaches to tuberculosis screening and the lived values of communities with a high burden of tuberculosis perhaps explains the limited success of active case finding as it is currently framed. Reorienting tuberculosis screening and care to include values of family and community connection may help improve both tuberculosis prevention and tuberculosis treatment outcomes. However, as such approaches are developed, it will be critical to consider people for whom family support is not available, ensuring they are not left behind [58]. This includes the deleterious effects of migration, urbanisation and precarious or hazardous work on access to support networks [61].

Whilst our intervention sought to broaden the scope of tuberculosis screening from the individual to include other members of tuberculosis-affected households, drivers of syndemics also include wider structural and social forces [62]. In order to effectively intervene on this or any syndemic, there is a need for coordinated, multi-sectoral action that intervenes on upstream determinants of tuberculosis in addition to direct individual or household-level interventions [1]. Here, the multiple needs of members of tuberculosis-affected households related not only to their health but also finances and education. Both participation in health screening and ability to engage with healthcare generally were shaped by upstream, contextual factors. In the tuberculosis context, poverty (exacerbated by the catastrophic costs of tuberculosis [63]) is of critical importance. This is amenable to interventions such as cash transfers, school fee waivers, or other forms of social protection, however to date the household-level and non-tuberculosis specific impacts of social protection interventions have been rarely considered [64,65]. Community support groups, social support and cultural resources, home visits, and mental healthcare have been proposed as essential components of syndemic interventions and are highly effective in the context of people living with HIV [66]. Future development of syndemic-oriented tuberculosis screening and services could have an expanded scope, incorporating these additional mechanisms.

Strengths of this study include a contextually relevant package of services, developed with community input and based on previous experience, with a mixed methods design that captured the perspective of participants. We importantly captured data on linkage to care after screening and on outcomes at 12 months, which are rarely measured in screening interventions, but are critical to understand potential impact. Use of realist evaluation theory enabled us to elucidate the ‘middle range’ theory of the intervention, thus informing its development and applicability across contexts [34].

An important limitation of this study is that it was nested within a research project and delivered at a central (government-hospital) site in an urban setting, with support for transport. It is likely that engagement with and outcomes of the intervention will differ when delivered in a more local context (for example local clinics). However, through elucidating the underlying mechanisms, we have gained generalisable insights into how these may be enabled or impeded by different intervention components. Importantly, we did not record the demographics nor captured the perspectives of people with tuberculosis or household contacts who were not willing to disclose their status to family members or participate in tuberculosis screening. These groups may differ from the people who did participate in screening in terms of burden of disease, whilst it is likely that the acceptability of the intervention and barriers to participation will be different. We did not perform a cost analysis, as this is also likely to differ as the intervention moves into a real-world setting. We did not include children under the age of 10 and therefore importantly, have not captured the needs of, or impact of an integrated intervention on, younger children. Further, our understanding of intervention mechanisms was restricted to an urban context: future work should explore how mechanisms differ across settings and include rural populations. As this study did not have a comparator (either standard-of-care or ‘tuberculosis screening only’) we are not able to explore how mechanisms and outcomes may be directly attributable to the integrated ‘health check’, rather than other elements of the intervention (such as compassionate staff and education and counselling about tuberculosis), or at how this intervention may increase uptake of tuberculosis screening among household contacts. In this study, a minority of tuberculosis household contacts received TPT (provided by government sector-services not by the study [67]) despite this being in stock in all clinics (as reported by clinic staff). Associative HIV stigma may contribute to low uptake of TPT, since TPT is dispensed from ART clinics. It is possible that, through similar mechanisms to those elucidated, our family-centred, integrated health check approach would have increased uptake of and adherence to TPT had this been included within the package of care. It is also important to note that our definitions of chronic diseases were based on point-of-care screening on a single day, which may lack specificity (e.g., for hypertension [68]) or sensitivity (e.g., for diabetes [69]). Whilst the lack of a comparator means it is not possible to determine how many of the conditions identified by the health check would have been otherwise detected through routine health services in the absence of this intervention, the inaccessibility of health services for tuberculosis-affected households (as revealed in our in-depth interviews, prior work [27] and lived experience in Zimbabwe) means this number is likely to be small. We also cannot determine how much the intervention contributed to participants achieving disease control at 12 months follow up, as compared to what would have occurred in its absence. Given the relatively low linkage to care for diabetes, it is surprising that 90% of people had ‘disease control’ at 12 month follow up. This may reflect the impact of lifestyle intervention and/or regression to the mean. Given follow up data were incomplete, it is also possible that participants who were more engaged with health services (and therefore were more likely to achieve disease control) were more likely to be retained in the study. Such evaluations will require larger-scale implementation research, for example as a cluster-randomised trial.

In conclusion, this study has provided evidence for and a theory of how integrated services, offered for free within systematic screening of tuberculosis household contacts, could improve the health of these vulnerable families. An integrated ‘health-check’ approach achieved high uptake, yield, and linkage to care and was found to be likely to result in beneficial outcomes through multiple mechanisms. This supports further evaluation in large-scale implementation studies within routine tuberculosis screening, including determination of cost-effectiveness. Our intervention however represents only one piece of the puzzle of syndemic care for tuberculosis-affected communities – improving the health and wellbeing of tuberculosis-affected communities demands co-ordinated multisectoral and multi-level action.

## Supporting information

S1 Appendix Supplementary methods and results. (DOCX)
