# Supplementary material for: Integrated health checks as a person-centred approach to systematic screening of household tuberculosis contacts: A realist-informed mixed-methods study
Source: PLOS Glob Public Health. 2025 Nov 3;5(11):e0005146. doi: 10.1371/journal.pgph.0005146 (PMC12582465; doi:10.1371/journal.pgph.0005146)
Supplement: S1 Appendix — (DOCX) [file pgph.0005146.s001.docx]

**Integrated health checks as a person-centred approach to systematic screening of household tuberculosis contacts: a realist-informed mixed-methods study**

**SUPPLEMENTARY MATERIALS**

[Supplementary methods 3](#_Toc199868469)

[Sample size 3](#_Toc199868470)

[Table A: Sample size calculations 3](#_Toc199868471)

[Table B: Template for intervention description and replication (TiDieR) checklist 3](#_Toc199868472)

[Table C: Consolidated criteria for reporting qualitative research (COREQ) checklist 4](#_Toc199868473)

[Considerations for inclusion of conditions in chronic disease screening 5](#_Toc199868474)

[Table D: Conditions selected for screening and accompanying justification 6](#_Toc199868475)

[Table E: Screening and referral procedures and assessment of linkage to care 7](#_Toc199868476)

[Figure A: Initial programme theory 9](#_Toc199868477)

[Recruitment to the integrated health check 9](#_Toc199868478)

[Table F: Timing of recruitment to the integrated health check within the ERASE-TB study 9](#_Toc199868479)

[Study site and support for transport 9](#_Toc199868480)

[Study team 9](#_Toc199868481)

[Intervention delivery (additional details) 10](#_Toc199868482)

[Follow up procedures 10](#_Toc199868483)

[Quantitative data collection 10](#_Toc199868484)

[Procedures for in-depth interviews and thematic analysis 10](#_Toc199868485)

[Topic guide for in depth interviews 11](#_Toc199868486)

[Table G: Characteristics of participants in in-depth interviews (N=28) 14](#_Toc199868487)

[Table H: Coding framework 16](#_Toc199868488)

[Supplementary results 20](#_Toc199868489)

[Healthcare seeking context & barriers 20](#_Toc199868490)

[Table I: Problems encountered when seeking care for health concerns among participants at recruitment to the health check (N=466)* 20](#_Toc199868491)

[Table J: Uptake and yield of screening among members of tuberculosis-affected households (N=467) 20](#_Toc199868492)

[Table K: Prevalence of chronic conditions by age strata and sex (N=467) 20](#_Toc199868493)

[Concentration of and interactions between chronic conditions 21](#_Toc199868494)

[Figure B: Tetrachoric correlation matrix illustrating correlation between pairs of conditions (N=467) 21](#_Toc199868495)

[Figure C: Intersection plot illustrating common disease dyads and triads among adults participating in the integrated health check (N=348) 23](#_Toc199868496)

[Table L: Characteristics of participants who did and did not link to care for hypertension 23](#_Toc199868497)

[Table M: Characteristics of participants who did and did not link to care for diabetes 23](#_Toc199868498)

[Table N: Change in individual components of SSQ 24](#_Toc199868499)

[Table O: EQ5D and mental health by whether or not people linked to care 24](#_Toc199868500)

[Healthcare seeking in the period after the integrated health check 24](#_Toc199868501)

[Table P: Costs incurred by participants in the 12 months following the integrated health check (N=398) 24](#_Toc199868502)

[Table Q: Key themes and illustrative quotes from thematic analysis 25](#_Toc199868503)

[References 29](#_Toc199868504)

# Supplementary methods

**Terminology**

We primarily use the term ‘households’ or ‘household members’ when referring to the people living in tuberculosis -affected households, who do not have tuberculosis themselves. We use the term ‘household contacts’ when discussing TB programming; reflecting the language used in this context. We also use the term ‘family’ or ‘family members’ when reporting on the findings of in-depth interviews, reflecting the framing made by the respondents. A household was defined as a grouping of people who reside under the same roof for at least three nights per week for the preceding four weeks, or who shared meals. Members of the same household may not be members of the same family (as is the case for ‘co-tenants’ or sub-lets, where two or more families each take a proportion of the rooms of a shared space) and, equally, members of a family may reside outside of the household (as is the case for people who work away from home). In Zimbabwe, it is common for families to include extended family members who reside across different households, areas of the country, or in other countries, but who continue to have key family roles, including providing financial support.

## Sample size

Whilst the sample size was determined by the primary aim of the parent study, we performed power calculation, in advance of recruitment, to demonstrate the adequacy for our aims. We intended to invite all 700 household contacts to participate. Table 1 illustrates sample sizes required across a range of potential yields of screening and degrees of precision. Based on observed data from participants recruited to ERASE-TB (the parent study) before19th January 2022 (n= 356 HHC and 152 index cases) the anticipated cluster size was 2.34 household contacts per household (in addition to the index case). Thirty percent of participants were anticipated to be under 18 years old.

The sample size required to estimate a proportion (i.e. yield) with confidence interval shown across a range of design effects of 1.15–1.3 (intra-cluster correlation coefficients = 0.05–0.1, cluster size = 2.34; accounting for 10% non-response) was calculated (Table A).

## Table A: Sample size calculations

| 95%CI +/- | | | 2% | | 3% | | 4% | |
| --- | --- | --- | --- | --- | --- | --- | --- | --- |
| ICC | | | 0.05 | 0.1 | 0.05 | 0.1 | 0.05 | 0.1 |
|  | |  | N required | | | | | |
| Percentage | 2% | | 221 | 235 | 99 | 105 | 56 | 59 |
|  | 5% | | 536 | 570 | 238 | 253 | 134 | 143 |
|  | 10% | | 1015 | 1079 | 451 | 480 | 254 | 270 |
|  | 20% | | 1804 | 1917 | 802 | 852 | 451 | 480 |
|  | 30% | | 2368 | 2516 | 1053 | 1119 | 592 | 629 |
| **Footnotes:** Green boxes indicate sample sizes that are met by the study population as was achieved. **Abbreviations:** 95%CI = 95% confidence interval, ICC = intra-cluster correlation coefficient, N = number. | | | | | | | | |

Table B: Template for intervention description and replication (TiDieR) checklist

| Item number | Item | Where located ** | |
| --- | --- | --- | --- |
|  |  | Primary paper  (line number) | Other ^†^ (details) |
|  | BRIEF NAME |  |  |
| 1. | Provide the name or a phrase that describes the intervention. | 1 |  |
|  | WHY |  |  |
| 2. | Describe any rationale, theory, or goal of the elements essential to the intervention. | 151 |  |
|  | WHAT |  |  |
| 3. | Materials: Describe any physical or informational materials used in the intervention, including those provided to participants or used in intervention delivery or in training of intervention providers. Provide information on where the materials can be accessed (e.g. online appendix, URL). | 186 | S1 pg 10 |
| 4. | Procedures: Describe each of the procedures, activities, and/or processes used in the intervention, including any enabling or support activities. | 186 |  |
|  | WHO PROVIDED |  |  |
| 5. | For each category of intervention provider (e.g. psychologist, nursing assistant), describe their expertise, background and any specific training given. | 186 | S1 pg 10 |
|  | HOW |  |  |
| 6. | Describe the modes of delivery (e.g. face-to-face or by some other mechanism, such as internet or telephone) of the intervention and whether it was provided individually or in a group. | 186 | S1 pg 10 |
|  | WHERE |  |  |
| 7. | Describe the type(s) of location(s) where the intervention occurred, including any necessary infrastructure or relevant features. | 186 | S1 pg 10 |
|  | WHEN and HOW MUCH |  |  |
| 8. | Describe the number of times the intervention was delivered and over what period of time including the number of sessions, their schedule, and their duration, intensity or dose. | 186 |  |
|  | TAILORING |  |  |
| 9. | If the intervention was planned to be personalised, titrated or adapted, then describe what, why, when, and how. | NA |  |
|  | MODIFICATIONS |  |  |
| 10.^ǂ^ | If the intervention was modified during the course of the study, describe the changes (what, why, when, and how). | NA |  |
|  | HOW WELL |  |  |
| 11. | Planned: If intervention adherence or fidelity was assessed, describe how and by whom, and if any strategies were used to maintain or improve fidelity, describe them. | NA |  |
| 12.^ǂ^ | Actual: If intervention adherence or fidelity was assessed, describe the extent to which the intervention was delivered as planned. | NA |  |

Table C: Consolidated criteria for reporting qualitative research (COREQ) checklist

| Item No | Guide Questions/Description | Reported on Page # |
| --- | --- | --- |
| Domain 1: Research team and reflexivity | | |
| Personal Characteristics | | |
| 1. Interviewer/ facilitator | Which author/s conducted the interview or focus group? | S1 pg 10 |
| 2. Credentials | What were the researcher’s credentials? E.g., PhD, MD | S1 pg 10 |
| 3. Occupation | What was their occupation at the time of the study? | S1 pg 10 |
| 4. Gender | Was the researcher male or female? | S1 pg 10 |
| 5. Experience and training | What experience or training did the researcher have? | S1 pg 10 |
| Relationship with participants | | |
| 6. Relationship established | Was a relationship established prior to study commencement? | S1 pg 10 |
| 7. Participant knowledge of the interviewer | What did the participants know about the researcher? e.g. personal goals, reasons for doing the research? | S1 pg 10 |
| 8. Interviewer characteristics | What characteristics were reported about the interviewer/facilitator? e.g. Bias, assumptions, reasons and interests in the research topic | NA |
| Domain 2: study design | | |
| Theoretical framework | | |
| 9. Methodological orientation and Theory | What methodological orientation was stated to underpin the study? e.g. grounded theory, discourse analysis, ethnography, phenomenology, content analysis | S1 pg 11 |
| Participant selection | | |
| 10. Sampling | How were participants selected? e.g., purposive, convenience, consecutive, snowball | Line 222 |
| 11. Method of approach | How were participants approached? e.g., face-to-face, telephone, mail, email | S1 pg 10 |
| 12. Sample size | How many participants were in the study? | S1 pg 11 |
| 13. Non-participation | How many people refused to participate or dropped out? Reasons? | S1 pg 11 |
| 14. Setting of data collection | Where was the data collected? e.g., home, clinic, workplace | S1 pg 11 |
| 15. Presence of non-participants | Was anyone else present besides the participants and researchers? | S1 pg 11 |
| 16. Description of sample | What are the important characteristics of the sample? e.g. demographic data, date | S1 table G |
| Data collection | | |
| 17. Interview guide | Were questions, prompts, and guides provided by the authors? Was it pilot tested? | S1 pg 10 |
| 18. Repeat interviews | Were repeat interviews carried out? If yes, how many? | Line 227 |
| 19. Audio/visual recording | Did the research use audio or visual recording to collect the data? | Line 229 + S1 pg 11 |
| 20. Field notes | Were field notes made during and/or after the interview or focus group? | Line 234 + S1 pg 11 |
| 21. Duration | What was the duration of the interviews or focus group? | S1 pg 11 |
| 22. Data saturation | Was data saturation discussed? | S1 pg 11 |
| 23. Transcripts returned | Were transcripts returned to participants for comment and/or correction? | S1 pg 11 |
| Domain 3: analysis and findings | | |
| Data analysis | | |
| 24. Number of data coders | How many data coders coded the data? | S1 pg 11 |
| 25. Description of the coding tree | Did the authors provide a description of the coding tree? | Table H |
| 26. Derivation of themes | Were themes identified in advance or derived from the data? | S1 pg 6 |
| 27. Software | What software, if applicable, was used to manage the data? | Line 251 |
| 28. Participant checking | Did participants provide feedback on the findings? | S1 pg 11 |
| Reporting | | |
| 29. Quotations presented | Were participant quotations presented to illustrate the themes/findings? Was each quotation identified? e.g., participant number | Table 4 |
| 30. Data and findings consistent | Was there consistency between the data presented and the findings? | Line 336 onwards |
| 31. Clarity of major themes | Were major themes clearly presented in the findings? | Line 336 onwards |
| 32. Clarity of minor themes | Is there a description of diverse cases or a discussion of minor themes? | Line 336 onwards |

## Considerations for inclusion of conditions in chronic disease screening

Conditions were included in the screening package based on: i) established associations with TB; ii) evidence to suggest high prevalence and/or under-diagnosis of disease in the local population (i.e. public health importance); iii) evidence of high value placed on screening in previous qualitative research in the local population; iv) availability of an accurate and acceptable screening test which can be implemented in the study setting, with results available on the same day (i.e. point of care) and v) availability of an appropriate local, accessible intervention for onward referral and treatment for people identified through screening (Table E). We also considered the need to ensure confidentiality in this household-based intervention, with this being one reason we opted for an audio-computer-assisted self-interviewing format; providing reassurance for participants that answers to sensitive questions could not be overheard. It is important to note, for future development of this intervention, that not all health screening may be suitable in this context. For example, we considered screening for sexually transmitted infections, given their high prevalence in Zimbabwe, but considered this not feasible due to issues around partner notification and confidentiality in the context of family members of visiting the screening site as a unit.

Table D: Conditions selected for screening and accompanying justification

| Condition | Justification |
| --- | --- |
| TB | TB HHC are high risk for TB; early diagnosis improves outcomes |
| HIV | 23% undiagnosed  Risk factor for TB progression; effective intervention to prevent TB (ART, TPT) |
| Malnutrition | Risk factor for TB  Risk factor for poor TB-related outcomes  Recommended by WHO^4^ |
| Visual impairment | Cause of social and economic hardship  Screening highly valued by community^5^ |
| Anaemia | Screening highly valued by community^5^ |
| Alcohol use disorder (age 14+) | Risk factor for TB and poor outcomes |
| Common mental health disorders (age 14+) | Associated with TB. Economic hardship and/stigma are risk factors. |
| Diabetes mellitus  (age 18+) | 90% undiagnosed^6^. Risk factor for progression for TB. Associated with poor TB outcomes. |
| Hypertension  (age 18+) | 75% undiagnosed^6^. Important public health cause of morbidity and mortality. |

**Abbreviations**: ART = anti-retroviral therapy; HHC = household contacts; TPT = TB preventive therapy; TB = tuberculosis; WHO = World Health Organization.

Table E: Screening and referral procedures and assessment of linkage to care

| Condition | Screening test(s) and definition of positive | Further assessment, treatment and care | Assessment of linkage to care | Definition of disease control at follow up |
| --- | --- | --- | --- | --- |
| Screening for all participants | | |  |  |
| HIV | HIV 1/2 blood based rapid test positive (including confirmatory test positive; as per national guidelines) | People newly screening positive or not on ART referred to primary care clinics for ART +/- IPT as per National guidelines. | Telephone calls to participants at 8-12 weeks after referral, questionnaire at 12 month follow up | HIV viral load <80 copies/uL |
| TB | WHO symptom screen +/- CXR abnormal triggers Xpert Mtb/Rif Ultra. TB diagnosed if Xpert Mtb/Rif Ultra positive or clinical diagnosis on basis of CXR and medical review | People diagnosed with TB referred to local TB services to initiate treatment. | Telephone calls to participants at 8-12 weeks after referral, questionnaire at 12 month follow up | Not assessed |
| Underweight | BMI <18.5 kg/m^2^ (among adults) or Z-score for age <-2 (among adolescents) | Food hampers (household-wide) provided by the study at baseline, 3 months and 6 months for all is any member of the household underweight.* | Log of disbursements of food hampers | BMI ≥18.5 kg/m^2^ (among adults) or Z-score for age ≥-2 (among adolescents) |
| Distance vision impairment | Peek Acuity app | Referral to ophthalmology clinic for assessment and for spectacles (with costs covered by the study) where applicable. | Ophthalmology clinic log including clients assessed and provided with glasses; questionnaire at 12 month follow up | Participants asked about whether they had and wore glasses regularly |
| Anaemia | Hb (Hemocue 301+; Hemocue), categorized using WHO age and sex-stratified definitions of anaemia | Anaemia is common and may be a non-specific finding; therefore, only those with severe anaemia (Hb <8g/dL) referred. People with lesser degrees of anaemia asked about relevant symptoms and counselled on diet, and referred if indicated. | Telephone calls to participants at 8-12 weeks after referral, questionnaire at 12 month follow up | Hb (Hemocue) above WHO age and sex-stratified thresholds for anaemia |
| Age-stratified screening | | |  |  |
| Adults (18+ years) | | |  |  |
| Diabetes | HbA1c ≥6.5% (A1c Care, SD Biosensor) | Undiagnosed diabetes or known diabetes but HbA1c ≥6.5% referred to chronic disease clinic; lifestyle management counselling | Records from study nurses attending chronic disease clinic with clients; questionnaire at 12 month follow up | HbA1c ≤6.5% (A1c Care, SD Biosensor) |
| Hypertension | BP ≥140/90mmHg (measured 3 times, 5 minutes apart as per WHO STEPs protocol | New finding of hypertension (lowest of 3 BP readings ≥140/90) referred to chronic disease clinic; lifestyle counselling. $2 given per client to support retesting of BP to confirm diagnosis prior to clinic appointment. | Records from study nurses attending chronic disease clinic with clients; questionnaire at 12 month follow up | BP <140/90mmHg |
| Adolescents (14+ years) and adults | | |  |  |
| Mental health | Shona Symptom Questionnaire >8 or red flags (ACASI) | Referral to counselling support unit (no costs incurred to participant) for telephone-based or on-site counselling | Counselling unit completed case report forms, returned to study for invoicing | Shona Symptom Questionnaire ≤8 and no red flags (ACASI) |
| Alcohol use disorder | AUDIT (ACASI) | Brief counselling and referral to counselling support unit for telephone-based or on-site counselling | Counselling unit completed case report forms, returned to study for invoicing | Not assessed |
| Older adults (40+ years) | | |  |  |
| Near vision impairment | Peek Acuity app | On-site provision of reading glasses | Log of glasses offered and provided to clients within the tablet screening tool | Participants asked about whether they had and wore glasses regularly |
| Abbreviations: ACASI = audio-computer assisted self-interview; AUDIT = alcohol use disorders identification test; BMI = body mass index, BP = blood pressure; CSU = counselling support unit; PHC = primary health clinic. * The intention at conception of the study was for participants to be referred to local social support services, however service mapping during intervention demonstrated these were fragmented and challenging to access; we therefore opted to provide a locally-appropriate food hamper directly to participants. | | | | |

## Figure A: Initial programme theory


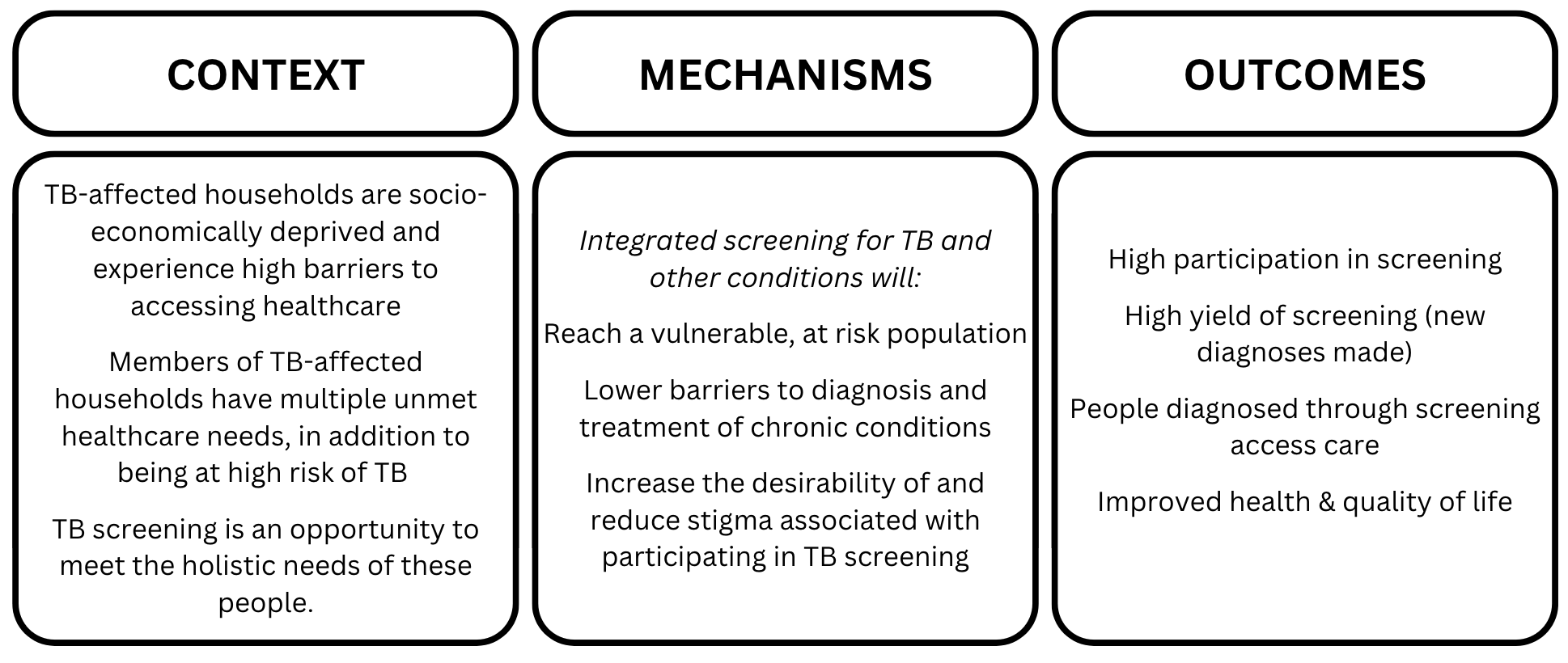


**Footnotes:** Prior to this study, our programme explanatory theory was that if people from tuberculosis-affected households are offered additional services, then they will be more willing to participate in tuberculosis screening because of greater perceived benefit, and will be diagnosed with important conditions earlier, enabling them to access treatment, and resulting in improved quality of life. Examining the key mechanism driving our intended outcomes, we expected that the motivation would be concern among members of tuberculosis-affected households for their own health and an eagerness to access services that may be difficult to access or not otherwise available. **Abbreviations**: TB = tuberculosis.

## Recruitment to the integrated health check

Recruitment to the main ERASE-TB study in Zimbabwe started on 2nd March 2021 and recruitment to the sub study started on 20th April 2022. As a result, 446 participants had already been recruited to the study. We allowed household members to participate at any of the baseline, six-month or 12-month study visits.

## Table F: Timing of recruitment to the integrated health check within the ERASE-TB study

| **Visit** | **N (%)** |
| --- | --- |
| Enrolment | 234 (50.1%) |
| +6 months | 78 (16.7%) |
| +12 months | 155 (33.2%) |

## Study site and support for transport

This study was based at a tertiary care facility which caters for referrals from the southern suburbs of Harare and elsewhere. Whilst most of our participants were recruited from the southern suburbs of Harare; some participants were recruited from surrounding satellite towns within the hospital’s catchment area. Although the study clinic was independent from the operations of the tertiary institution, we selected this location for reasons of logistic feasibility, enabling us to evaluate a large number of participants at a single location, as required for the assessment of diagnostic accuracy of TB tests.^1^ Public transport in Zimbabwe is based on a network of buses and small minivans, however during COVID-19 the Government of Zimbabwe suspended this network as an infection prevention measure, with the unintended consequence being widespread transport challenges. We therefore elected to collect participants from their homes (or a mutually agreed location) and bring them to the site.^2,3^

## Study team

The intervention was delivered by a nurse (TM; male) and research assistant (initially SS, then later EM; both female) employed as part of the study team. ETM (public health professional and nurse; male) and CJC (physician and researcher; study principal investigator; female) provided oversight and support, including clinical advice as needed. TM has a diploma in nursing (Zimbabwe) and experience in government-sector primary care delivery before joining the study team and conducted clinical tests, communicated results and provided counselling. SS and EM are research assistants with experience in mixed methods research. Their primary responsibilities were data collection, linkage to care and follow up; however, the team worked collaboratively with shared roles. The team were trained on all specific study procedures (e.g. screening tests and questionnaires), principles of tuberculosis screening and management, principles of person-centred care, and motivational interviewing methods.

## Intervention delivery (additional details)

The intervention was delivered face-to-face. To maximise airflow and maintain COVID-19 infection, prevention and control precautions, study procedures were delivered in tents, erected on a concreted area near a small clinic room, with (intermittent) power and water (used for storage, charging devices, etc). Participants often attended the study as family groups; however, each participant was seen individually and family members asked to sit some distance away aimed to ensure privacy from other family members. Risks of stigmatization of participants through inadvertent disclosure of their health status to other members of their family or community were discussed and measures were taken to mitigate this risk; no such events related to study participation were reported to the study team. Health and safety considerations for researchers were mapped, and were managed in accordance with institutional policies at the Biomedical Research and Training Institute.

## Follow up procedures

Follow up data varied by condition and included direct reporting from providers, telephone contact with participants 8-12 weeks after referral, and a questionnaire at follow up (Table E).

Participants were followed up 12 months after participation in screening. Disease ‘control’ was assessed for diabetes, hypertension, anaemia, and mental health by repeating the tests used for screening. Participants with vision impairment were asked whether they had glasses and wore these regularly. HIV viral load testing was performed for participants with HIV, either immediately, with feed-back of results, for those who wished to have a viral load test, or anonymously on stored samples. People who reported that they were living with a health condition at follow up were asked to complete an interviewer-administered structured questionnaire about time and costs associated with accessing health care (adapted from the TB patient cost survey), barriers to healthcare and sources of support.

## Quantitative data collection

Quantitative data were collected via electronic tablets at screening visit and 12 month follow up, synced to an Open Data Kit server hosted at the London School of Hygiene & Tropical Medicine and analysed using R (version 4.3.1).

## Procedures for in-depth interviews and thematic analysis

Additional details of qualitative data collection are provided here in accordance with the consolidated criteria for reporting qualitative research (checklist: Table C).^7^ Interviews were conducted by MN (Female, MSc) and MT (Female, MSc), research assistants employed as part of the study but who were not involved in intervention implementation. Both have postgraduate degree-level training in social science research, and previous experience in in-depth interviewing in Zimbabwe. CJC trained the interviewers in the aims and rationale of the study and provided an overview of the early quantitative findings. The topic guide (below) was collaboratively developed by CJC, MT and MN, with input from KK and JD, piloted with two participants, and iteratively refined based on emerging data. Potential participants were purposively sampled and approached face-to-face (for example, at the time of participating in the intervention) or via phone; at which time the interviewers explained the objectives of the research. Interviews were conducted at the study site, the research team’s offices or participant homes, according to participant convenience and preference. Only the interviewer and interviewee were present during interviews. All interviews were audio-recorded and transcribed within a week of the interview taking place. The final sample size was determined by saturation in thematic analysis. No repeat interviews were conducted, and transcripts were not returned to participants for review.

In total, 28 participants were interviewed (14 men and 15 women; median age 35.5 years [range 15–67 years) between 4^th^ May 2023 and 13^th^ January 2024 (Table G). No-one refused to participate in interviews.

Analysis was based in a grounded theory approach. The coding framework was developed through independent coding of the first four interviews by MN, MT and CJC, followed by discussion and definition of codes derived from the data (Table H). It was iteratively refined throughout the analysis. The realist evaluation framework was selected based on it providing a good fit to the emergent themes. All interviews were then coded by MT and CJC, with regular meetings to discuss emergent themes. Individual participants did not provide feedback on the findings, but these were presented to the community advisory board and intervention team for feedback, which has informed the final interpretation and narrative.

## Topic guide for in depth interviews

Introduction:

We have invited you to come for an interview because you have recently taken part in the ERASE-TB study. The aim of this interview is to help deepen our understanding of your experiences of taking part in ERASE-TB, your preferences when being tested for TB and other medical problems, and how this fits into your wider experiences of health, illness and accessing medical services, including your experiences with other healthcare providers.

We are very interested in your experiences and opinions, whether these are positive or negative. Hearing both positive and negative views is important for our research, so please speak freely and honestly. The information you share with us will be confidential, even from the nurses and research assistants you saw as part of ERASE-TB.

If you do not want to answer any question, that is OK, please just say so. We expect the interview to take 1 hour but if you would like to stop at any time, please just say so.

Note: key questions are in **bold**, sub questions / prompts in *italics*

Context:

1. Could you tell me about yourself?
   1. Can you describe your family and home setting / your work/schooling?
   2. How would you describe your health?
   3. Can you tell me about any health problems you have currently, or have had in the past?
2. What is your experience of getting healthcare usually in your community?
   1. What was good / bad about these experiences? Why was this good / bad?
   2. What services are available and easy to access? Why is this the case?
   3. Are services available for free or for a fee? If for a fee, is it affordable to you?
3. What did you know about TB and its impact in your community before you came to be part of ERASE-TB?
   1. Who gets TB? How it is transmitted? What happens to someone with TB?
   2. Can you please explain on what you have heard about TB treatment?
   3. Is TB a big or a small problem in your community? Why do you think that is the case?
4. Who was the person who had TB recently in your family?
   1. For example, what was their relationship to you?
   2. How did you come to know about their illness and diagnosis?
5. When you found out that [index case] had TB, what were your thoughts and feelings?
   1. Why did you feel this way?
   2. Was there anything you were afraid of? (Specifically, fears around TB)
   3. Did you do anything differently as a result of these fears?
   4. Looking back now, do you still feel this way, or have these thoughts/feeling/fears changed since that time? How so?

Participating in ERASE-TB

1. What do you think are the reasons for conducting the ERASE-TB study?
2. Why did you decide to participate in the ERASE-TB study?
   1. What were you hoping for; was there anything that appealed to you (and why)?
   2. Did you have any concerns about your own health at the time (what and why)?
   3. Was there anything that put you off (and why); what made you decide to participate despite these worries?
3. Please describe what happened when you came to ERASE-TB and how you found this?
   1. Can you describe the tests that were done as part of the study and how those were for you?
   2. What were the things that you liked or didn’t like about the tests? Why so?
   3. Was this what you were expecting; if not, how did it differ? How do you feel about that?
   4. Is there anything you particularly liked about the study? What was it? Why did you like that?
   5. Is there anything you did not like or would have liked to be done differently? What was it? Why did you want that to be different?
4. In ERASE-TB, we are testing for TB and other diseases as well. Were you tested for anything else other than TB?
   1. Ask specifically about TB, diabetes, hypertension, vision, mental health, HIV.
   2. If not, why not? Were these tests offered to you?
   3. Were you aware that these tests for other disease would be included before coming to the study? Was this something that affected your decision to participate?
   4. What did you understand about why the study was offering tests for TB and for other diseases?
   5. How did you feel about being tested for TB? Why did you feel that way?
   6. How did you feel about being tested for those other conditions? Why did you feel that way?
5. Did you learn new anything about your health through participating in ERASE-TB, maybe that you did not know before? What did you learn?
   1. What were your feelings on receiving that information? Why did you feel that way?
   2. Was there anything that wasn’t clear?
   3. Was there anything you didn’t get to know that you would like to know?
   4. If you were told you had a medical problem, did you believe this result straight away, or did you have any doubts? Why was this the case? How do you feel about the result now?
6. If they learned anything (Q9): What did you do with this information: can you tell me the story of what happened next? How did you feel during that time?
   1. Did you go and see someone for further information or treatment?
   2. Where did you go first? Why did you go to that place? What happened when you were there?
   3. Have you also been to other places for information/treatment? Where did you go and why? What happened at those places?
   4. Who do you feel supported you during this time? How did they help?
   5. Can you describe for me and specific worries / fears / frustrations that you had during that time?
   6. If you were found to have a health problem: Have you been given any treatment for [the health problem]? How did the treatment go / is the treatment going?
   7. Was there anything you would have liked to be able to do with the information you got about your health but couldn’t? Why couldn’t you do these things?
7. Can you compare the experience you had with ERASE-TB to experiences of healthcare elsewhere? How was it similar or different?
   1. Were any differences positive or negative? Why so?
   2. What impact did any differences have on you? (e.g. on willingness to participate, satisfaction with services, knowledge, etc)
8. In ERASE-TB I understand you are asked to come back multiple times.
   1. What do you understand about why they ask you to do that?
   2. Has it been easy or difficult for you to come for multiple visits? Why was it easy / difficult?
9. Looking at everything that was done as part of the ERASE-TB study, what is your opinion on the study? Why do you feel that way?
   1. Are there any features of the study that you found particularly good? What and why?
   2. Are there any features of the study that you did not like? What and why? What are your suggestions on improving this?

Preferences

Now I would like you to imagine you were the one designing a health programme like ERASE-TB, which was providing services to families (contacts) where someone has TB.

1. Where and how should this service be delivered? Why should it be like this?
   1. Where should this service be based?
   2. Who should be the staff delivering the service?
   3. Would people be willing to come to the service? What would motivate people come to the service? What would put them off coming?
2. What diseases or services should be included? Why should these services be offered?
   1. Why do you suggest including those specific diseases / services?
   2. What would the benefit of such a programme be?
3. Are there any things that you think should not be include in such a programme (and why)?
4. What do you think are the things that might stop such a programme from being possible? If it was possible, what would stop it from working well?

Impact of TB and disease screening & close

1. Looking back at everything that has happened since [index case] got TB, how has your life changed?
   1. Probe into: financial, social (family relationships, relationships with neighbours / in community), psychological etc.
2. That is the last of my questions. Is there anything else you would like to talk about with me?

Table G: Characteristics of participants in in-depth interviews (N=28)

|  | **N participants** |
| --- | --- |
| Age, years | 35.5 years (range 15–67) |
| Female | 15/28 |
| Employed | 8/28 |
| Relation to person with TB |  |
| Spouse | 7 |
| Parent / aunt / uncle / grandparent | 2 |
| Other* | 12 |
| Health conditions** |  |
| HIV | 6 |
| Hypertension | 6 |
| Mental health screen positive | 3 |
| Previous TB | 3 |
| **Footnotes**: * Other includes child, niece, nephew, sibling or cousin. **Conditions with group size >2 shown. | |

Table H: Coding framework

| Theme | Sub-themes | Contributing codes |
| --- | --- | --- |
| Context | | |
| Chronic financial insecurity and psychosocial stress is exacerbated by the TB episode which results in loss of income | Chronic financial insecurity and psychosocial stress | Need to provide for the family |
|  | Unstable, risky or un-employment | Mining, Need to travel to get work, South Africa, Vendor work |
|  | TB results in loss of income causing anxiety around how to pay for essential needs including food |  |
|  | Coping strategies include borrowing, sale of assets, and decisions about children’s education | Impacts on schooling, Lack of food |
|  | Need to generate income is a barrier to accessing health services | Limited time available to seek healthcare; Time off work |
| Highly resource-constrained health system results in, clinic fees, stock-outs, and overstretched, sometimes uncaring, staff; however community-based health facilities are available and offer a range of services | Accessibility of services | Confusion, Distance to clinics |
|  | Clinics lack resources, they are busy, and staff are overstretched | Advice may differ from different clinics; Not enough staff; Slow process; Queues; Attitude of staff; Medication stock outs and prescriptions |
|  | Delays in accessing healthcare may be related to experiences at health facilities | Embarrassment; Fear; Lack of 'care'; Continuity of care; Lack of control; Lack of help |
|  | Costs of healthcare |  |
|  | It is necessary to pay for healthcare - often not affordable | Consultation fees; Costs of medication; Flexibility applied when people cannot afford to pay is variable; Need to save |
|  | Other costs associated with accessing care include transport charges |  |
|  | Fragmented services | Multiple trips or places |
|  | In general, people reported positive experiences having accessed care | TB and HIV services; Joint TB and HIV care |
|  | Multiple pathways to health care | Coexistence of biomedical and non-biomedical; Apostolic church; Variety of public and private healthcare providers |
|  | Services available at government sector clinics include HIV and TB treatment services, antenatal or maternity, child vaccinations, STIs, cervical cancer screening and medicines dispensing for chronic conditions |  |
| Strong family-based support structures exist. These facilitate care seeking and support treatment adherence | 'Others' may stigmatise or exclude PWTB or family | Stigma around TB (and HIV); Having to move house; Non-acceptance, rejection or isolation; Perceptions of 'others'; Disclosure and non-disclosure |
|  | Families also experience other challenging events | Deaths in the family |
|  | Family structures and role of extended family | Extended family; Shared houses |
|  | God and religion as a source of support |  |
|  | The family comes together to provide multifaceted support to the person with TB (a support system) | Taking measures to prevent TB (or not); Acceptance of (the person with) TB; Disclosure of health status; Ability to support; Concern; Relief; To protect family; Neccessary for recovery from TB; Advice and reassurance; Family members 'triggering' TB care seeking; Practical support; Caring (for people with TB); Food; Money; Monitoring; To enable people to take medication; Psychosocial support; Putting the person who is sick first; Responsibility (adolescents) |
|  | Supporting someone with TB is challenging | TB treatment; Appetite; Default; Hope; Recovery; Taking medication |
| Few reliable sources of information about TB, resulting in lack of understanding, myths and misconceptions, and fear | In some families, multiple people have had or have TB | Knowing that others have had TB aids acceptance; Recurrent TB |
|  | TB symptoms may not be immediately recognised as such or acted on | Delayed health seeking |
|  | Family members feelings about TB range from fear to hope, and are modulated by knowledge about the disease | Fear of death; Fear of infection; Fear of transmission; Fear; Lack of reliable information; Hope; TB is usually fatal; Death; Early treatment for TB improves outcomes; Anxiety; Emotional pain; Shock |
|  | Perceived causes of TB, including certain behaviours, can lead to blame towards the person with TB | TB is usually due to an underlying 'cause'; Witchcraft can cause TB |
|  | HIV and TB are closely related to each other |  |
|  | TB has long-lasting and multiple impacts on affected people and families | Disruption vs continuation; Impact of TB illness on ability to work; Mental health |
|  | Coping strategies - borrowing, sale of assets, decisions about education | Education; Loss of income |
|  | Family members of someone with TB perceive themselves as being at high risk of being infected with or developing TB, this leads to fear | Children are at particular risk; Precautions are needed to prevent TB transmission in households; Not sharing food or utensils with someone with TB; TB is transmissible and therefore contacts are at risk; Isolation |
|  | TB not perceived as a big issue in the community |  |
| Familiarity with the rationale for screening and benefits of early diagnosis from messaging around HIV | HIV - familiarity with screening |  |
|  | Early treatment results in better outcomes |  |
|  | Knowing your status is a 'good thing' | Knowing your status |
|  | Individual health concerns at the time of being invited to screening | Assumptions about health |
| Mechanisms |  |  |
| The high perceived benefit of screening outcompeted other priorities. This benefit included the ‘opportunity’ to be tested for TB, and access other services that are usually inaccessible. | High anticipated benefit promotes participation, and outcompetes other priorities | Anticipated benefit; 'Pull' factors; Desire for good health; Risk of non-participation; To be tested; Assistance; Benefits of testing; Accurate diagnosis; Consequences of not testing; Reassurance; Safety; Prevention |
|  | Being tested for TB was the primary motivation to participate in screening | Early diagnosis; Fear; Prevent transmission |
|  | Being tested for HIV was a motivator - but sometime also associated with fear |  |
|  | Barriers to accessing health services include lack of confidentiality and community stigma, particularly for TB and HIV | Barriers to participating in screening; Reluctance; Willingness to participate in screening; Continuing with work or school; Competing priorities; 'Push' factors; Miss school; Lack of time |
|  | Need to raise awareness | Lack of understanding; Make people aware of the study; Need for community buy in; Word of mouth |
|  | Expectation of integrated services - or expected it to only be about TB | Expectations |
| Members of TB-affected households want their family to be healthy, and often emphasise this over their own health needs | 'Route in' to screening is through the index case | Experience of index case can influence decisions to participate; Come with family |
|  | Integrated services were highly acceptable | Ability to 'help'; Direction from God; Need for services; Treatment; Accessibility of services; Local services; Rural areas; Services close by; Care for the whole person; Integrated services; Mental health; HIV testing |
|  | The opportunity for free, accessible services that people would otherwise not be able to afford was valued ('a rare opportunity') | Need for services; Positive experience; Reduced 'work' of accessing care |
|  | Receiving results | Acceptance; Anticipation; Denial; Happiness; Same day; Stress |
| Compassionate staff delivering person-centred, integrated care makes people feel respected and valued | Approach of staff | Accomodating; Caring; Doing 'as expected'; Friendly; Knowledgeable; Professionalism; Role modelling; Staff take time; Comfortable; Privacy; Routine care vs ERASE-TB |
| Improved understanding of TB, HIV and NCDs through education and counselling about TB and non-communicable diseases increased capacity for self-management and care seeking when needed, and reduced self-stigma | Advice and education | Ability to educate others; Counselling on NCDS; On TB prevention |
|  | Education and counselling on TB and its treatment equipped family members to provide care and support for people with TB | Overcoming fear |
|  | Lifestyle and diet changes | Making changes to reduce the risk of getting TB |
| Multiple considerations around engagement in care, including internal understanding of illness and motivation to seek care, as well as substantial health system challenges. | Barriers to service accessibility mean that ongoing engagement in healthcare was challenging and support for this was valued | Challenges navigating the health system - multiple places; Lack of TB medications means frequent visits; Process of going to clinic; Time taken at clinic; Cost of consultation; Cost of medications; Unable to buy medications; Need to go to the hospital; Screening should be linked to 'assistance', but not taken as a given; Peer support |
|  | Individual understanding of illness and concerns around the course of illness and treatment deterred people from accessing care | Adherence to medication; Anticipated path of treatment; Comparison of HIV and other conditions; Fear of side effects; Pill burden; |
|  | People often perceive high blood pressure as being the result of circumstance (for example due to the stress of caring for an unwell family member), rather than a chronic condition – even when it is repeatedly high across healthcare encounters. | Most people with hypertension were not on treatment; Motivation; Understood causes of illness |
| Members of TB-affected households also have urgent and fundamental non-health needs |  | Schooling; (lack of) employment; Travelling for work; Caring for relatives |

# Supplementary results

## Healthcare seeking context & barriers

Among 467 participants, at the time of recruitment, 10% (n=48) reported seeking healthcare in the previous 12 months for at least one medical problem. Mostly, this related to acute health problems (n=29), HIV (n=8) or a chronic health problem (n=7). Of people who sought healthcare, most (n=30) attended a public health clinic or hospital (n=7). Cost was the most encountered problem when attending health facilities (n=113; 24% participants; Table I).

## Table I: Problems encountered when seeking care for health concerns among participants at recruitment to the health check (N=466)*

|  | Not a problem | A small problem | A big problem | Decline to answer/ Don’t know |
| --- | --- | --- | --- | --- |
| Cost of attending health facility | 251 (54%) | 102 (22%) | 113 (24%) | 0 (0%) |
| Distance to health facility | 365 (78%) | 60 (13%) | 40 (9%) | 1 (0%) |
| Gaining permission | 394 (85%) | 52 (11%) | 19 (4%) | 1 (0%) |
| Having someone to accompany me to clinic | 384 (82%) | 62 (13%) | 20 (4%) | 0 (0%) |
| Industrial action | 319 (68%) | 87 (19%) | 48 (10%) | 12 (3%) |
| Opening hours | 351 (75%) | 78 (17%) | 31 (7%) | 6 (1%) |
| Quality of services | 324 (70%) | 77 (17%) | 53 (11%) | 12 (3%) |
| Services available | 327 (70%) | 83 (18%) | 47 (10%) | 9 (2%) |
| Way in which services are provided | 321 (69%) | 69 (15%) | 64 (14%) | 12 (3%) |
| Footnotes:* The question posed to participants was: “Many different factors can affect how people get medical advice or treatment for themselves. When you are sick and want to get medical advice or treatment, is each of the following a big problem or not a big problem for you?”. These data were missing for one participant. | | | | |

## Table J: Uptake and yield of screening among members of tuberculosis-affected households (N=467)

|  | **Screened*** | **Declined screening** | **Screen negative** | **Screen positive** | **Known** | **Prevalence** |
| --- | --- | --- | --- | --- | --- | --- |
| TB | 459 (98.3%) | 8 (1.7%) | 454 (98.9%) | 5 (1.1%) |  | 5 (1.1%) |
| HIV | 452 (96.8%) | 15 (3.2%) | 380 (84.1%) | 7 (1.5%) | 65 (14.4%) | 72 (15.9%) |
| Underweight | 466 (99.8%) | 1 (0.2%) | 436 (93.6%) | 30 (6.4%) |  | 30 (6.4%) |
| Anaemia | 467 (100%) |  | 404 (86.5%) | 63 (13.5%) |  | 63 (13.5%) |
| Diabetes | 346 (99.4%) | 2 (0.6%) | 314 (90.8%) | 23 (6.6%) | 9 (2.6%) | 32 (9.2%) |
| Hypertension | 342 (98.3%) | 6 (1.7%) | 223 (65.2%) | 60 (17.5%) | 59 (17.3%) | 119 (34.8%) |
| Common mental health disorders | 380 (93.6%) | 26 (6.4%) | 260 (68.4%) | 117 (30.8%) | 3 (0.8%) | 120 (31.6%) |
| Visual impairment | 460 (98.5%) | 7 (1.5%) | 340 (73.9%) | 81 (17.6%) | 39 (8.5%) | 120 (26.1%) |
| **Footnotes**: * Screening for diabetes and hypertension was not indicated among people aged <18 years (n = 119 [25.5%]) and screening for common mental health disorders was not performed among people <14 years (n = 61 [13.1%]). Individuals in which screening was not indicated are excluded from the table. | | | | | | |

Table K: Prevalence of chronic conditions by age strata and sex (N=467)

| **Characteristic** | **Overall**  N = 467 | **Male**  N = 168 | **Female**  N = 299 | **<18 years**  N = 119 | **18-39 years**  N = 211 | **40+ years**  N = 137 |
| --- | --- | --- | --- | --- | --- | --- |
| HIV (n=459) | 80 (17%) | 19 (11%) | 61 (21%) | 1 (0.9%) | 36 (17%) | 43 (32%) |
| Diabetes (n=346) | 32 (9.2%) | 6 (5.4%) | 26 (11%) | – | 11 (5.3%) | 21 (15%) |
| HbA1c, % (n=346) | 5.80  (5.50, 6.20) | 5.80  (5.40, 6.18) | 5.80  (5.50, 6.20) | – | 5.70  (5.40, 6.00) | 6.00  (5.60, 6.30) |
| Hypertension (N=342) | 119 (35%) | 34 (31%) | 85 (37%) | – | 39 (19%) | 80 (60%) |
| Common mental health disorders (N=380) | 120 (32%) | 33 (27%) | 87 (34%) | 11 (20%) | 59 (30%) | 50 (40%) |
| SSQ score (N=380) | 4.0 (2.0, 8.0) | 3.0 (1.0, 7.0) | 5.0 (2.0, 8.0) | 2.5 (1.0, 4.8) | 4.0 (2.0, 8.0) | 6.0 (4.0, 9.0) |
| Red flags present (N=380) | 53 (14%) | 19 (16%) | 34 (13%) | 6 (11%) | 24 (12%) | 23 (18%) |
| Underweight (N=466) | 30 (6.4%) | 19 (11%) | 11 (3.7%) | 7 (5.9%) | 16 (7.6%) | 7 (5.1%) |
| Anaemia (N=467) | 63 (13%) | 17 (10%) | 46 (15%) | 31 (26%) | 15 (7.1%) | 17 (12%) |
| Haemoglobin, g/dL | 13.8 (13.0, 15.0) | 15.0 (13.7, 16.0) | 13.5 (12.8, 14.3) | 13.5 (12.9, 14.5) | 14.0 (13.1, 15.4) | 13.7 (12.9, 14.8) |
| Vision impairment (N=460) | 120 (26%) | 38 (23%) | 82 (28%) | 7 (6.1%) | 30 (14%) | 83 (61%) |
| Distance vision impairment | 74 (16%) | 23 (14%) | 51 (24%) | 6 (5%) | 21 (10%) | 47 (34%) |
| Near vision impairment | 58 (42%) | 15 (34%) | 43 (46%) | – | – | 58 (42%) |
| ≥1 condition (N=467) | 310 (66%) | 104 (62%) | 206 (69%) | 45 (38%) | 134 (64%) | 131 (96%) |
| Multimorbidity (N=467) | 165 (35%) | 40 (24%) | 125 (42%) | 12 (10%) | 51 (24%) | 102 (74%) |
| N conditions (N=467) | 1.0 (0.0, 2.0) | 1.0 (0.0, 1.0) | 1.0 (0.0, 2.0) | 0.0 (0.0, 1.0) | 1.0 (0.0, 1.0) | 2.0 (1.00, 3.0) |
| **Footnotes**: presented as n (%) and median (interquartile range). * Screening for diabetes and hypertension was not indicated among people aged <18 years (n = 119) and screening for common mental health disorders was not performed among people <14 years (n = 61); near vision impairment was only screened for among participants aged 40+ years. Red flags on the Shona Symptom Questionnaire (SSQ) are defined as answering yes to either of “Did you sometimes feel like committing suicide?” or “Did you sometimes see or hear things others could not see or hear?”. | | | | | | |

## Concentration of and interactions between chronic conditions

On an individual level, strongest correlations between chronic conditions were observed between vision impairment and hypertension (0.51), vision impairment and diabetes (0.36) and hypertension and diabetes (0.34; Figure B). Underweight was negatively correlated with diabetes and hypertension (-0.32 and -0.30) and positively correlated with common mental health disorder (0.36), whilst tuberculosis was correlated with HIV (0.44) and anaemia (0.33). The most common chronic disease dyads were hypertension/vision impairment (23/467 participants), hypertension/common mental health disorder (13/467 participants) and HIV/common mental health disorder (10/467 participants); the most common triad was hypertension/vision impairment/common mental health disorder (10/467 participants; Figure C).

## Figure B: Tetrachoric correlation matrix illustrating correlation between pairs of conditions (N=467)


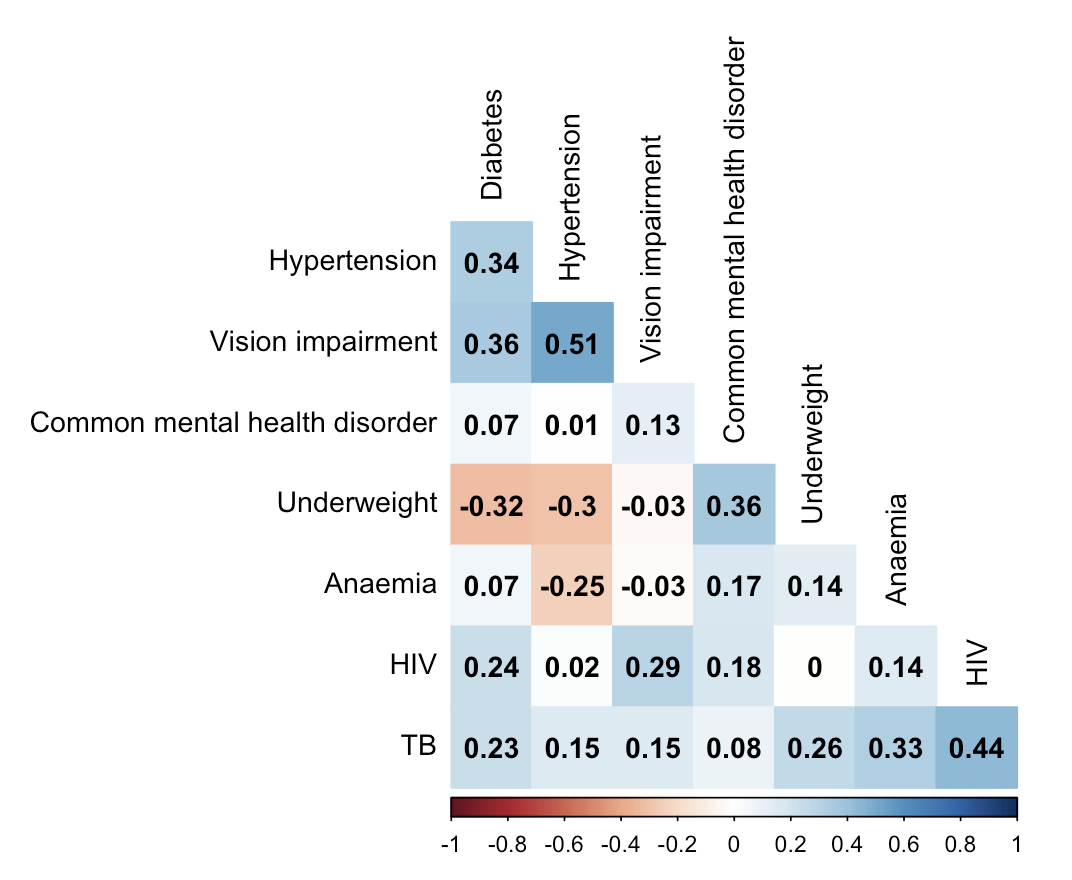


Figure C: Intersection plot illustrating common disease dyads and triads among adults participating in the integrated health check (N=348)


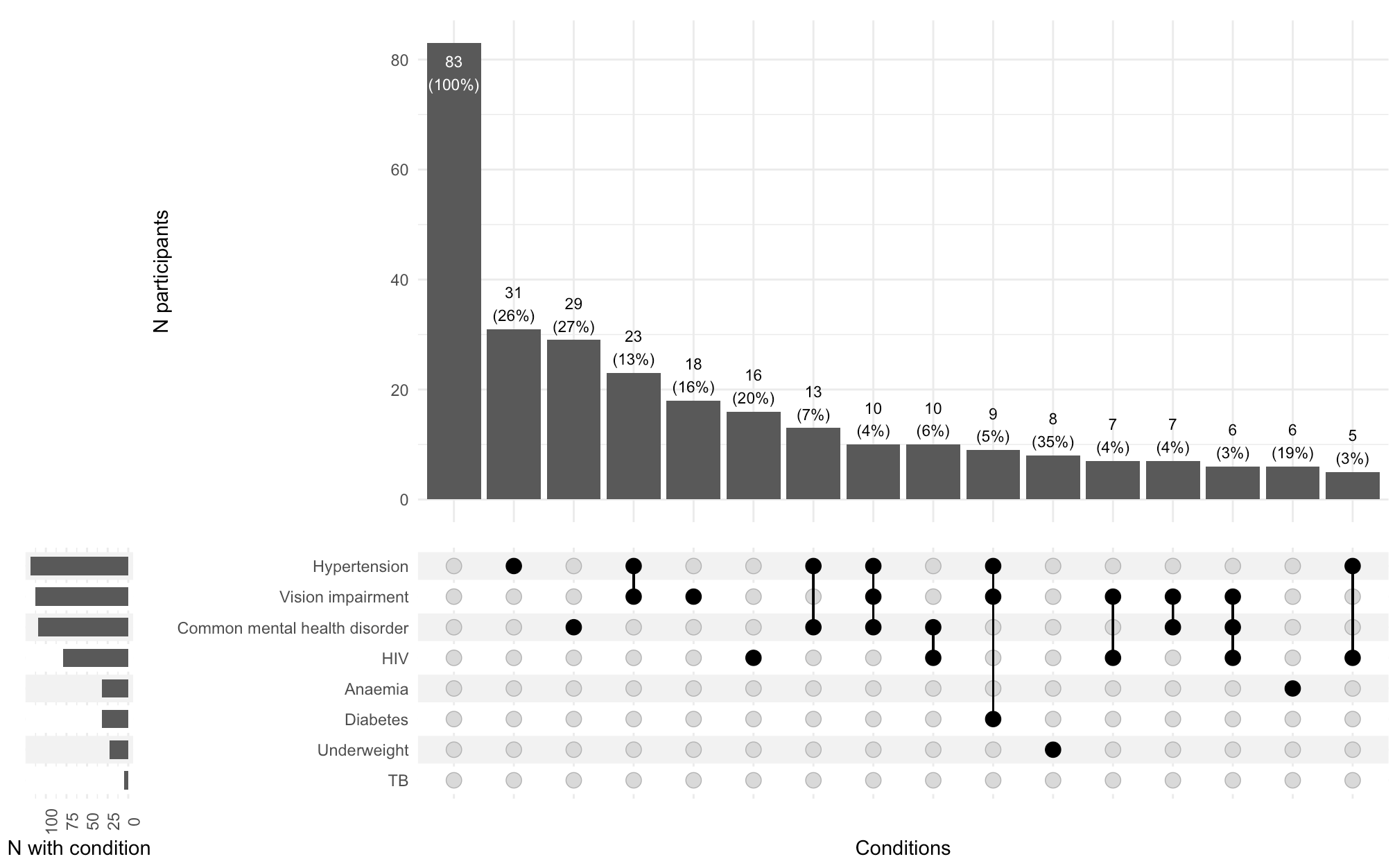


**Footnotes**: Intersections are exclusive (i.e. individuals are only represented in the graph once) and only intersections containing at least 5 individuals are shown. The bottom sub-panel shows the included chronic conditions (y axis), with the left section indicating the overall number (N) participants with each and the right section showing the specific disease combinations (represented by black circles). The top sub-panel shows intersection size, i.e. the number of participants with each disease combination. Percentages are relative to the potential size of the intersection.

Table L: Characteristics of participants who did and did not link to care for hypertension

|  | Linked to care for hypertension | |  |
| --- | --- | --- | --- |
| ****Characteristic**** | **No** (N = 56) | **Yes** (N = 41) | **p-value** |
| Age, years | 44 (34–51) | 49 (39–54) | 0.075 |
| Female | 37 (66%) | 31 (76%) | 0.3 |
| Known HTN | 18 (32%) | 18 (44%) | 0.2 |
| Grade 2 HTN | 22 (39%) | 15 (37%) | 0.8 |
| Systolic BP, mmHg | 144 (140–161) | 146 (140–165) | 0.4 |
| Diastolic BP, mmHg | 94 (88–100) | 95 (90–107) | 0.3 |
| Footnotes: presented as median (IQR) or number (%). P values are from Wilcoxon rank sum test (continuous variables); or Fishers exact test (categorical variables). | | | |

Table M: Characteristics of participants who did and did not link to care for diabetes

|  | Linked to care for diabetes | |  |
| --- | --- | --- | --- |
| Characteristic | **No** (N=22) | **Yes** (N=9) | **p-value** |
| Age, years | 38 (24–48) | 49 (47–52) | 0.033 |
| Female | 18 (82%) | 7 (78%) | >0.9 |
| Known diabetes | 1 (4.5%) | 3 (33%) | 0.063 |
| HbA1c, % | 6.75 (6.60–7.03) | 8.30 (6.70–10.60) | 0.13 |
| Footnotes: presented as median (IQR) or number (%). P values are from Wilcoxon rank sum test (continuous variables); or Chi-squared test (categorical variables). | | | |

## Table N: Change in individual components of SSQ

|  | **Baseline**  N = 380 | **Follow up**  N = 360 | **Percentage difference** |
| --- | --- | --- | --- |
| Did you find yourself sometimes failing to concentrate? | 171 (45.0%) | 121 (33.6%) | -11.4% |
| Were there times when you felt life was so tough you cried or wanted to cry? | 211 (55.5%) | 104 (28.9%) | -26.6% |
| Were you frightened by trivial things? | 74 (19.5%) | 53 (14.7%) | -4.8% |
| Did you sometimes see or hear things others could not see or hear?* | 29 (7.6%) | 19 (5.3%) | -2.4% |
| Was your work lagging behind? | 131 (34.5%) | 59 (16.4%) | -18.1% |
| Did you feel you had problems deciding what to do? | 178 (46.8%) | 102 (28.3%) | -18.5% |
| Did you sometimes fail to sleep or did you lose sleep? | 179 (47.1%) | 142 (39.4%) | -7.7% |
| Did you have nightmares or bad dreams? | 149 (39.2%) | 130 (36.1%) | -3.1% |
| Was your stomach aching? | 169 (44.5%) | 127 (35.3%) | -9.2% |
| Did you sometimes feel like committing suicide?* | 33 (8.7%) | 21 (5.8%) | -2.9% |
| Did you lose your temper or get annoyed over trivial matters? | 154 (40.5%) | 129 (35.8%) | -4.7% |
| Did you sometimes think deeply or think about many things? | 181 (47.6%) | 156 (43.3%) | -4.3% |
| Did you feel run down (tired)? | 173 (45.5%) | 109 (30.3%) | -15.2% |
| Were you generally unhappy with the things you were doing each day? | 93 (24.5%) | 62 (17.2%) | -7.3% |
| **Footnotes**: SSQ only completed among participants aged ≥14 years. * Indicate ‘red flag’ questions | | | |

Table O: EQ5D and mental health by whether or not people linked to care

| **Timepoint** |  | **Baseline** | | **Follow up** | |
| --- | --- | --- | --- | --- | --- |
| **Linked to care for at least one condition** | | **No** | **Yes** | **No** | **Yes** |
|  | | **N=236** | **N=144** | **N=221** | **N=139** |
| SSQ | Score | 3 (1–6) | 8 (4.75–10) | 2 (0–5) | 4 (1.5–7) |
|  | Positive screen | 34 (14.4%) | 86 (59.7%) | 29 (13.1%) | 38 (27.3%) |
|  |  | **N=225** | **N=149** | **N=196** | **N=134** |
| EQ5D-5L | Value (median) | 0.90 (0.86–0.90) | 0.87 (0.83–0.90) | 0.90 (0.85–0.90) | 0.86 (0.82–0.90) |
|  | No difficulties (value) | 157 (69.8%) | 74 (49.7%) | 118 (65.9%) | 65 (48.9%) |
| **Footnotes**: Presented as median (interquartile range) or number (percentage). SSQ completed by all consenting participants aged ≥14 years and EQ5D-5L completed by all consenting participants aged ≥16 years. | | | | | |

## Healthcare seeking in the period after the integrated health check

At baseline, 46/466 people (10%) reported having sought help with their health in the previous 12 months. Among 398 people followed up, 152 (38%) had attended a clinic or hospital in the previous 12 months. Table P summarises the costs incurred by participants when seeking care in the follow up period. Whilst small numbers limited comparisons, costs appeared higher among people with hypertension compared to HIV (e.g. an overall median total expenditure [excluding income loss] of 3USD among people with HIV [IQR 0–28SUSD] vs 7USD [0–48USD]).

The most reported sources of support for managing chronic conditions were friends/family (n=43; 48%), followed by pharmacies (n=13) and the internet/social media (n=12).

## Table P: Costs incurred by participants in the 12 months following the integrated health check (N=398)

| Characteristic | No cost incurred | Cost incurred | Median (IQR) expenditure in USD among people with a cost incurred |
| --- | --- | --- | --- |
| Direct costs | 269 | 129 (32%) | 28 (11, 65) |
| Admission | 387 | 11 (2.8%) | 250 (55, 375) |
| Diagnostic tests | 363 | 35 (8.8%) | 25 (15, 80) |
| Clinic fees | 303 | 95 (24%) | 10 (5, 20) |
| Medications | 294 | 104 (26%) | 20 (10, 50) |
| Indirect costs | 324 | 74 (19%) | 5 (2, 15) |
| Food | 362 | 36 (9.0%) | 5 (2, 21) |
| Transport | 334 | 64 (16%) | 5 (2, 9) |
| Income loss | 384 | 14 (3.5%) | 45 (20, 480) |
| Total costs | 254 | 144 (36%) | 28 (11, 76) |
| Total costs including income loss | 254 | 144 (36%) | 28 (11, 97) |
| Abbreviations: IQR = interquartile range; USD = United States Dollars | | | |

Table Q: Key themes and illustrative quotes from thematic analysis

| **Theme** | **Subthemes / Quotes** |
| --- | --- |
| **Context** |  |
| Tuberculosis-affected households are financially insecure and have limited healthcare access and engagement | “I only reached O level but I couldn’t manage to write all the subjects because of lack of money (34M, no diagnoses)”  “Not everyone can afford [to go to the clinic] because some people are employed whilst others are not. They will not be able to afford it.” (20F, no diagnoses)  “The way you are treated, it is very difficult in healthcare facilities. On some days the service may be good and on some days it can be bad. It depends on the person’s mood, some people bring their moods from home to the workplace.” (46F, HIV & HTN) |
| Tuberculosis diagnosis affects the whole family, but strong family-based support structures often exist | “I had to sell most of the things that I had [when I developed TB]. I had to sell my car. I am the breadwinner so at the time that I fell sick, I needed medical attention, and I could no longer go to work. The only thing I could sell was my car so the children could go to school, and I could get medical attention (38M, no diagnoses)”  “Our brother [who died of TB] is the one who would fend for the family but now we just have to do it by ourselves. Everyone must contribute (43M, previous TB)”  “I got help from my brothers. They helped me [with money and groceries] until [my husband] recovered… (42F, HIV)”  “There was no one [who helped me]. It was just me and the children assisting each other… It was very difficult to accept [my husband being diagnosed with TB], I will not lie. People say different things…. They say many things, such as if someone is diagnosed with TB it means they are HIV positive. This makes life difficult. To continue living at the house you were living before becomes impossible. You will be forced to look for another house because the situation will not be good. People will not be comfortable around you and they will distance themselves” (39F, HIV & CMD) |
| Members of TB-affected households have a felt need for health screening and services | **Familiarity with the rationale for screening and benefits of early diagnosis from messaging about HIV**  “It is good because we could be infected [with TB] and not know about it. So if we have regular tests, we will know where we stand (15F, no diagnoses)”  There are situations where people are living with sicknesses that they do not know about and get to realise those diseases later. And yet all this time, it seemed as if the person was OK (34M, no diagnoses)’  ‘Blood sugar, HIV, BP, and many others… eyesight… I was tested for mental health… I was actually very happy because I was tested for other diseases I have never been tested for… I like knowing my health status… I learned that I am fit and do not have any ailments. It is better than to live with suspicion (21M, no diagnoses)’  **Few reliable sources of information about TB, resulting in lack of understanding, myths and misconceptions, and fear**  “The TB I heard of is the one spread through sexual intercourse. I’m not sure. (42F, HIV)”  “I did not know much about TB at that time and I still know very little about it. However, I thought that were was no difference between one who has TB and another who has AIDS. It is only now I am beginning to see there is a difference, but I am still unsure of the effects of TB, I am not really sure how it is transmitted (21M, no diagnoses)”  “I knew that one can contract TB from dust and that people who work in mines or smoke are at high risk (15F, no diagnoses)”  “We thought, was he smoking cigarettes, did he do it when we did not see? Where did he get it because he never worked at a place that he could get it. (40F, HTN, asthma, vision impairment)”  “There could also be an element of African spiritual attacks causing the pain, because they work hand in hand with TB”  “Most people say that it is a very painful illness and it is rare for a person to survive after being diagnosed with TB (15F, no diagnoses)” |
| Health needs compete with other fundamental priorities | “It was after [the index case] was seriously sick [that] we went to the hospital because at first we were reluctant because of the expected bills, but then we got help from [a neighbour]” (34M, no diagnoses)  “By assisting the unemployed people to get jobs, to give medicines to those in need, to rehabilitate those who are going astray… You should give us jobs” (43M, previous TB)  “Do you not have others that can help with the other things… like school fees, as for me, I want my kids to have a good education, but right now, they do not have” (47F, vision impairment) |
| Poor continuity of care and adherence to therapies for chronic conditions. | **Substantial barriers to accessing care exist, including lack of awareness, confidentiality and stigma, finances and geographic factors**  “I think [sometimes people] fear what others in the community think. Some people live in communities where people discuss each other’s lives daily. They will be afraid that people will be gossiping in the community, or start avoiding them.” (15F, no diagnoses)  “I use public transport [to collect my medication]. It is 50c one way… I cannot walk the distance because my chest feels heavy. I will not manage it” (39F, HIV & CMD)  “They said my BP was very high and they referred me to Harare hospital, however, you know the hospital fees are high so I told them I could not afford it…” (67M, HIV, HTN, previous stroke)  “You can pay consultation fee which is $10 for adults. The hope is that from that $10 you will be able to get some medication only to be told there are no medicines in the clinic pharmacy and you will have to go and buy from another pharmacy, for which you may not have money. So you will be forced to go home because there is no more money” (46F, HIV & HTN)  **Individual understanding of illness and concerns around the course of illness and treatment influenced whether and how people accessed care**  “I used to work in a mine, we always worked with torches and at times slept with them on so I quickly understood that my eyes might have been a problem… I thought to myself, maybe my current situation is causing my high BP. I look after sick people at home and there is no way the mind will be calm in such a situation” (42M, HTN & vision impairment)  “There may come a time when I wake up and I am no longer able to work. That means I will no longer be able to purchase the medication… [My relatives] do not have money. They actually need assistance from me. If I fall sick then all will be lost…” (40F, HTN) |
| **Mechanisms** |  |
| Family members encouraged people with TB to engage in care | “[My husband] started to have cough,,, and sweating at night and losing weight. And I would advise him to go and get tested but you know how it is with men… we would argue about going to the clinic but then he would just go and buy pills. Until I called my mother in law, she is the one that then told him to go and get tested. (26F, HIV)” |
| Education and counselling on TB and its treatment equipped family members with the knowledge, skills and capacity to care for people with TB | “What helped me was the counselling we received. Now I know that TB is treatable… Now I am able to counsel other people who come and ask about my husband’s sickness. If they want to know the process, I tell them and I offer counselling because I know that TB is treatable – as long as you follow the regimen and complete the course, you will live” (39F, HIV & CMD)  “[Participating in the study] brought change because I understand I must continue providing [the index case] support to take his medication so he recovers. I learned that I should… be compassionate, just like the people in this place” (46F, HIV & HTN) |
| Members of TB-affected households wanted their family to be well | ‘If I was to get infected, the TB would be detected early, that is what I liked about [the study]… my family would be safe, especially those of us who spent more time with [the index case]. (40F, HIV)’  ‘I was so worried but I was happy for the programme… I was hurt because I thought I would become blind yet I have kids that need to be taken care of… I am worried that with time I might yet go blind (47F, vision impairment) |
| Accessible, integrated services increase anticipated benefit and promote participation, whilst compassionate staff delivering person-centred, integrated care made people feel respected and valued, increasing trust in services | “When we get here, we get tested for different diseases. However, when you go to the private doctor, they only treat you for what you complain of. They ask you what the problem is. When I say, I have tonsillitis, they ask you to open your mouth and they check. If needs be, they will inject you without checking for anything else. They just inject you and give you pills and that is it” (28F, no diagnoses)  ‘[The programme] is good because it reduces the burden on those that do not have adequate financial resources. Nowadays it is cash up front. If you have a headache, cash first, in order to get assistance (34M, no diagnoses)’  ‘I came here today and I am not feeling well, I have a cough. They can give me pills for that. This is different to the clinic because they simply write you a prescription and tell you to go and buy. Sometimes you do not have the money to buy (39F, HIV & CMD)’  “The way you are handled speaks volumes on your health and even your mind… because at some of our regular hospitals, we might have given up way back because of how they speak to patients… [At the study] they always speak in a nice way without any harsh tones. They are good people and they speak with you until you understand and they do not force anything on you…” (40F, HTN, asthma, vision impairment)  “There is a girl [in the house] who is 13 years old now, she never got tested… her and my husband never got tested because the times were clashing, because of work and school” (40F, HTN, asthma, vision impairment) |
| Improved access to healthcare enables engagement, but challenges remain | **Education and support provides motivation and ability to prioritise health**  “We reduced both smoking and alcohol” (36M, previous TB, CMD and vision impairment)  “I reduced my salt intake and [they said] that I should exercise regularly” (42M, HTN & vision impairment)  “I was told by someone to stop taking heavy foods (carbohydrates) and fizzy drinks, especially Pepsi…. She also said I must eat soft sadza in small portions. I am still doing that to this day” (39F, HTN)  **Convenience and cost of services**  ‘I would like it to be done in all the areas… maybe in the area where we live so that everyone can get tested… maybe at the school? (23F, no diagnoses)’  ‘If possible I would ask that these services should be provided in many places including rural areas. There are many people who are sick but do not know about you, they do not know how to find you, or that they can be assisted (31M, no diagnoses)  “What happened with the BP was that they gave me tablets for a month and they said that if I finish the pills I should start buying for myself. I bought them for 2 months but thereafter I did not have money to continue paying for the pills… if I was staying in [Town A] and working, I would be able to buy my medicine” (42M, HTN & vision impairment)  **Fear of taking treatment**  I would be lying if I spoke of medication. They only told me that my BP is high and I should go on treatment. I did not [go to the clinic]… I am afraid of taking medication… I was scared… I heard that if you do not take the medication, once you are on treatment, you can collapse and be out for the whole day and only come to later when you take the medication (laughs)… There are times that I feel numb on my left side. I do not know if that is caused by BP or another illness that is coming to me.” (39F, HTN) |
| **Abbreviations**: CMD = common mental health disorder, F = female, HTN = hypertension, M = male. | |

# References

1. Marambire, E. T. *et al.* Early risk assessment in paediatric and adult household contacts of confirmed tuberculosis cases by novel diagnostic tests (ERASE-TB): protocol for a prospective, non-interventional, longitudinal, multicountry cohort study. *BMJ Open* **12**, e060985 (2022).

2. Mazorodze, W. & Ndawana, E. State monopoly on urban transport system and human (in)security in Harare during the COVID-19 pandemic. *African Security Review* (2022).

3. Muronzi, C. Zimbabwe’s bus shortage is making commuters miserable. *Al Jazeera* https://www.aljazeera.com/economy/2021/7/2/zimbabwes-bus-shortage-is-making-commuters-miserable.

4. World Health Organization. *Guideline: Nutritional Care and Support for Patients with Tuberculosis*. https://apps.who.int/iris/handle/10665/94836 (2013).

5. Marambire, E. T. *et al.* Evaluation of a comprehensive health check offered to frontline health workers in Zimbabwe. *PLOS Global Public Health* **4**, e0002328 (2024).

6. Calderwood, C. J. *et al.* Prevalence of chronic conditions and multimorbidity among healthcare workers in Zimbabwe: Results from a screening intervention. *PLOS Global Public Health* **4**, e0002630 (2024).

7. Tong, A., Sainsbury, P. & Craig, J. Consolidated criteria for reporting qualitative research (COREQ): a 32-item checklist for interviews and focus groups. *International Journal for Quality in Health Care* **19**, 349–357 (2007).
